# Outcomes of the arterial switch for transposition during infancy using a standardized approach over 30 years

**DOI:** 10.1093/icvts/ivad070

**Published:** 2023-05-10

**Authors:** Nigel E Drury, Shafi Mussa, John Stickley, Oliver Stumper, Adrian Crucean, Rami Dhillon, Anna N Seale, Phil Botha, Natasha E Khan, David J Barron, William J Brawn, Timothy J Jones, Collaborators†, Paul A Miller, Ashish Chikermane, Tarak Desai, Chetan Mehta, Vinay K Bhole, Milind P Chaudhari, Michael Harris, Simon P McGuirk

**Affiliations:** Department of Paediatric Cardiac Surgery, Birmingham Children’s Hospital, Birmingham, UK; Institute of Cardiovascular Sciences, University of Birmingham, Birmingham, UK; Department of Paediatric Cardiac Surgery, Birmingham Children’s Hospital, Birmingham, UK; Department of Paediatric Cardiac Surgery, Bristol Royal Hospital for Children, Bristol, UK; Department of Paediatric Cardiac Surgery, Birmingham Children’s Hospital, Birmingham, UK; Department of Paediatric Cardiology, Birmingham Children’s Hospital, Birmingham, UK; Department of Paediatric Cardiac Surgery, Birmingham Children’s Hospital, Birmingham, UK; Institute of Cardiovascular Sciences, University of Birmingham, Birmingham, UK; Department of Paediatric Cardiology, Birmingham Children’s Hospital, Birmingham, UK; Institute of Cardiovascular Sciences, University of Birmingham, Birmingham, UK; Department of Paediatric Cardiology, Birmingham Children’s Hospital, Birmingham, UK; Department of Paediatric Cardiac Surgery, Birmingham Children’s Hospital, Birmingham, UK; Department of Paediatric Cardiac Surgery, Birmingham Children’s Hospital, Birmingham, UK; Department of Paediatric Cardiac Surgery, Birmingham Children’s Hospital, Birmingham, UK; Division of Cardiovascular Surgery, Hospital for Sick Children, Toronto, Canada; Department of Surgery, University of Toronto, Toronto, Canada; Department of Paediatric Cardiac Surgery, Birmingham Children’s Hospital, Birmingham, UK; Department of Paediatric Cardiac Surgery, Birmingham Children’s Hospital, Birmingham, UK; Institute of Cardiovascular Sciences, University of Birmingham, Birmingham, UK

**Keywords:** Transposition, Arterial switch, Survival, Reintervention

## Abstract

**OBJECTIVES:**

The aim of this study was to describe the early and late outcomes of the arterial switch for transposition.

**METHODS:**

A single-centre retrospective cohort study was conducted to assess the early and late outcomes of arterial switch performed during infancy using a standardized institutional approach between 1988 and 2018, compared by morphological groups.

**RESULTS:**

A total of 749 consecutive patients undergoing arterial switch during infancy were included, 464 (61.9%) with intact septum, 163 (21.8%) with isolated ventricular septal defect and 122 (16.3%) with complex transposition with associated lesions, including 67 (8.9%) with Taussig–Bing anomaly. There were 34 early deaths [4.5%, 95% confidence interval (CI) 3.1–6.1] with only 10 (2.6%) early deaths since 2000. Complex morphology (odds ratio 11.44, 95% CI 4.76–27.43) and intramural coronary artery (odds ratio 5.17, 95% CI 1.61–15.91) were identified as the most important risk factors for 90-day mortality. Overall survival was 92.7% (95% CI 90.8–94.6) at 5 years and 91.9% (95% CI 89.9–94.1) at 20 years; in hospital survivors, there were 15 (2.1%) late deaths during a median follow-up of 13.7 years. Cumulative incidence of surgical or catheter reintervention was 16.0% (95% CI 14.5–17.5) at 5 years and 22.7% (95% CI 21.0–24.0) at 20 years; early and late reinterventions were more common in the complex group, with no difference between the other groups.

**CONCLUSIONS:**

Using a standardized approach, the arterial switch can be performed with low early mortality, moderate rates of reintervention and excellent long-term survival. Concomitant lesions were the most important risk factor for early death and were associated with increased risk of late reintervention.

**Figure:**
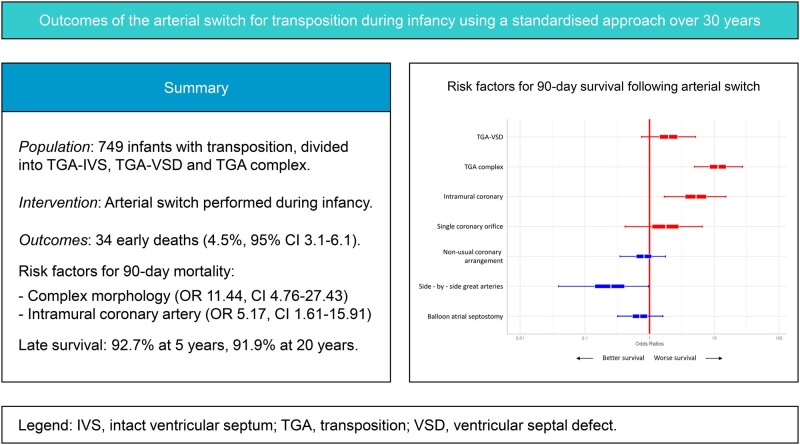

## INTRODUCTION

Transposition (TGA) is the most common cyanotic congenital heart defect presenting in neonates and without surgical intervention, long-term survival is rare [1, 2]. Since its introduction by Jatene in 1975 [3], the arterial switch has significantly improved the outcomes of children with TGA and has become the procedure of choice. Advances in antenatal detection and perioperative management [4], along with technical refinements including the Lecompte manoeuvre [5] and use of medially hinged trap-door incisions for coronary transfer [6], have led to low early mortality and excellent long-term survival [7–10]. Although technically demanding, the standardized procedure is reproducible and, within a mentoring framework, can be taught to newly appointed surgeons without compromising outcomes [11].

Morphological variations, such as ventricular septal defect (VSD), obstruction in the ventricular outflow tract or aortic arch and certain coronary artery patterns, increase the complexity of repair and have been associated with increased early mortality [12–15], but the impact on late outcomes remains uncertain. We conducted a retrospective analysis of infants with TGA treated with arterial switch at a single institution using a standardized approach over 30 years, to determine the impact of morphological variations on early and late survival and reintervention.

## PATIENTS AND METHODS

### Ethics statement

This study was registered with Birmingham Women’s and Children’s Research & Development office (BWC/LA/Drury/10, 04 October 2021) and in accordance with UK National Research Ethics Service guidance, neither individual informed consent nor formal research ethics committee review was required as the study was undertaken by the direct clinical care team using information previously collected in the course of routine care.

### Study population

All patients undergoing arterial switch during infancy at Birmingham Children’s Hospital, UK, between January 1988 and December 2018 were included. Patients over 1 year of age at arterial switch and those who underwent an alternative corrective procedure for complex TGA were excluded (see Supplementary Material).

### Operative technique

Our approach is to perform arterial switch in all patients with TGA, where it is technical feasible, within the first 2 weeks of life, unless there is an unrestricted VSD in which case we may leave longer to allow growth. In patients presenting up to 2 months, we still perform early arterial switch, with postoperative extracorporeal life support if required [16]; beyond 2 months, we would consider initial pulmonary artery banding, but this is now rare in the UK.

The surgical technique for arterial switch used by all surgeons at our institution remained consistent throughout the study period [11]; for a detailed description, see Supplementary Material. In brief, the ascending aorta was transected and the coronary arteries excised with a generous cuff of aortic sinus tissue and mobilized. The resultant defects were repaired with untreated autologous pericardium as a single patch. The main pulmonary artery was transected at the same level as the aorta and coronary artery buttons relocated to medially hinged trap-door incisions using an open technique to construct the proximal neoaorta [6]. Intramural coronary arteries were mobilized with generous cuffs of aortic wall, taking down the valve commissure or laying opening the ostia as required. If unable to transfer as described above, a pericardial hood technique was used, with a bovine pericardium or homograft patch to augment the receiving aortic sinus, incorporating the intramural cuff with minimal mobilization [17]. Lecompte manoeuvre was performed whenever possible [5], the neoaorta reconstructed, the heart re-perfused and reconstruction of the neopulmonary artery completed whilst rewarming with the heart beating. Additional procedures were performed to treat associated anomalies as required, a left atrial line was placed routinely and primary sternal closure was undertaken whenever feasible, or on the intensive care unit (ICU) as a delayed procedure, usually within 24–48 h.

### Clinical variables and follow-up

Data were obtained from patient records and institutional databases, reviewed and validated. Morphological data were collected from detailed descriptions in the operative records and the cohort divided into 3 groups:

- TGA-IVS: TGA with intact ventricular septum (IVS), including those in whom any VSD was deemed haemodynamically insignificant and therefore not closed,
- TGA-VSD: TGA with one or more isolated VSDs and
- Complex TGA: associated anomalies such as aortic arch obstruction/interruption, ventricular outflow tract obstruction or Taussig–Bing.

Coronary artery pattern was described by sinus of origin, according to the Leiden convention [18], and course around the great arteries [19]; Yacoub classification [20] was noted where possible.

Date of arterial switch was set as baseline for follow-up. Early death and early reintervention were defined as occurring within 30 days. Potential risk factors were estimated for 90-day mortality. For late deaths, data were obtained from the UK Office for National Statistics; cause of death was obtained from hospital notes and/or post-mortem examination.

### Statistical analysis

Analysis was performed using R version 3.6. Continuous variables are presented as median with interquartile range (IQR). Categorical variables are presented as frequencies and percentages. Comparisons between morphological groups were made using the Kruskal–Wallis tests for continuous variables, or Pearson's chi-squared test for categorical variables. Significance testing was two-sided with significance at *P* < 0.05.

Mortality was tracked using hospital attendance and UK Office for National Statistics national tracing service. Patients lost to follow-up were censored at the time last known to be alive. All-cause mortality was estimated using the Kaplan–Meier method and comparisons made using the log-rank test. Event rates for reinterventions were estimated using cumulative incidence function with death as the competing risk. Patients were assessed as free from reintervention only if being followed up locally, with out-of-region patients censored when last seen. To estimate risk factors for all-cause mortality, a logistic regression model was developed using a Bayesian method in R BRMS [21], with 90-day all-cause mortality as the outcome using sceptical prior distributions (see Supplementary Material). 95% compatibility intervals were derived from the posterior distribution and presented. The first author and statistician had full access to all data and take responsibility for its integrity and analysis.

## RESULTS

### Patients

**Figure 1:**
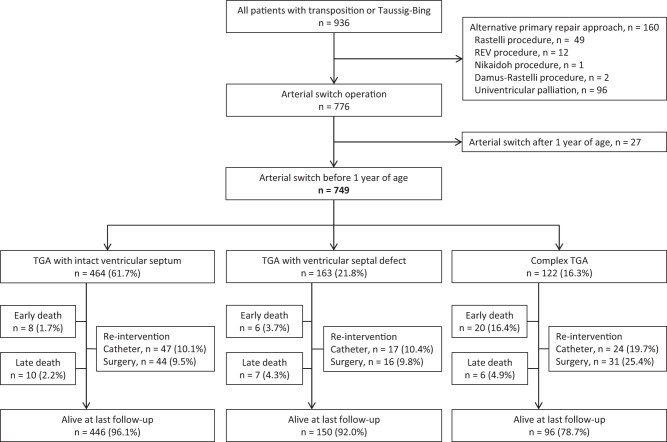
Flow diagram of children undergoing surgery for transposition at our institution. REV: Réparation à l’Etage Ventriculaire; TGA: transposition.

During the study period, 776 patients underwent arterial switch, of whom 749 were under 1 year of age and included in the analysis (Fig. 1). Since 1988, the arterial switch has been our procedure of choice for TGA and other operations only performed when this approach was deemed unsuitable (see Supplementary Material).

### Morphology

Of 749 infants undergoing arterial switch, 464 (61.9%) were diagnosed with TGA-IVS, 163 (21.8%) with TGA-VSD and 122 (16.3%) with complex TGA, including 67 (8.9%) with Taussig-Bing. Baseline characteristics and operative data are summarized in Table 1. Coronary artery anatomy had the usual origin (1LCx-2R), course and branching pattern in 507 (67.7%) patients, originated from a single orifice in 37 (4.9%) and followed an intramural course in 36 (4.8%). Non-usual coronary patterns were more frequent in the complex group (62, 50.8%, *P* < 0.001), with similar prevalence in the TGA-IVS and TGA-VSD groups (27.6% and 31.9%, respectively, *P* = 0.31). There was no difference between groups in the frequency of an intramural coronary artery (*P* = 0.23). Side-by-side great arteries were more common in complex TGA (*P* < 0.001) and typically associated with non-usual coronary patterns (*P* < 0.001). Detailed descriptions of the coronary patterns, including origin, course and branching, and associated early mortality are shown in Supplementary Material.

**Table 1:** Patient characteristics and operative data, by morphological group

Characteristic	Overall, *n* = 749	TGA-IVS, *n* = 464 (61.9%)	TGA-VSD, *n* = 163 (21.8%)	Complex TGA, *n* = 122 (16.3%)	*P-*Value
Age (days), median (IQR)	8 (5-16)	7 (5-12)	12 (6.5-23)	10.5 (5-39)	<0.001
Weight (kg), median (IQR)	3.4 (3.0-3.8)	3.4 (3.0-3.8)	3.4 (3.0-3.7)	3.4 (3.1-3.9)	0.40
Male, *n* (%)	531 (70.9)	327 (70.5)	120 (73.6)	84 (68.9)	0.65
Associated anomalies, *n* (%)					<0.001
VSD	305 (40.7)	33 (7.1) ^a^	163 (100)	109 (89.3)	<0.001
LVOTO	9 (1.2)	0	0	9 (7.4)	<0.001
RVOTO	23 (3.1)	0	0	23 (18.9)	<0.001
Coarctation of the aorta	86 (11.5)	0	0	86 (70.5)	<0.001
Interrupted aortic arch	10 (1.3)	0	0	10 (8.2)	<0.001
Taussig–Bing	67 (8.9)	0	0	67 (54.9)	<0.001
Coronary artery origins, *n* (%)^b^					<0.001
1LCx-2R	514 (68.6)	339 (73.1)	112 (68.7)	63 (51.6)	
Usual position in sinuses (A)	507 (67.7)	336 (72.4)	111 (68.1)	60 (49.2)	
Adjacent to commissure (C)	7 (0.9)	3 (0.6)	1 (0.6)	3 (2.5)	
1l-2CxR (D)	108 (14.4)	66 (14.2)	19 (11.7)	23 (18.9)	
1RL-2Cx (E)	60 (8.0)	24 (5.2)	16 (9.8)	20 (16.4)	
1R-2LCx (E)	4 (0.5)	1 (0.2)	1 (0.6)	2 (1.6)	
Sinus 1 only, including 1RLCx	17 (2.3)	10 (2.2)	2 (1.2)	5 (4.1)	
Sinus 2 only, including 2LCxR ^c^	46 (6.1)	24 (5.2)	13 (8.0)	9 (7.4)	
Intramural coronary origin(s), *n* (%)	36 (4.8)	24 (5.2)	4 (2.5)	8 (6.6)	0.23
Single coronary orifice (B),^b^ *n* (%)	37 (4.9)	16 (3.4)	11 (6.7)	10 (8.2)	0.048
Aorta-PA alignment, *n* (%)					<0.001
Aorta anterior and left of PA	30 (4.0)	23 (5.0)	3 (1.8)	4 (3.3)	
Aorta anterior to PA	459 (61.3)	311 (67.0)	99 (60.7)	49 (40.2)	
Aorta anterior and right of PA	218 (29.1)	124 (26.7)	51 (31.3)	43 (35.2)	
Side by side	42 (5.6)	6 (1.3)	10 (6.1)	26 (21.3)	
Balloon atrial septostomy, *n* (%)	532 (71.0)	395 (85.1)	99 (60.7)	38 (31.1)	<0.001
Additional procedure, *n* (%)					
VSD closure	269 (35.9)	0	163 (100)	106 (86.9)	<0.001
Aortic arch repair	84 (11.2)	0	0	84 (68.9)	<0.001
Relief of outflow tract obstruction	31 (4.1)	0	0	31 (25.4)	<0.001
Neo-PA banding, VSD not closed	3 (0.4)	0	0	3 (2.5)	N/A
LIMA-LAD coronary bypass graft	1 (0.1)	0	1 (0.6)	0	N/A
Other procedure	6 (0.8)	0	0	6 (4.9)	N/A
CPB time (min), median (IQR)	129 (107-155)	115.5 (101-136)	140 (122-160.5)	164 (149-208)	<0.001
AXC time (min), median (IQR)	76 (62-95)	67 (58-81)	85 (73-98.5)	110.5 (97-129)	<0.001
DHCA used, *n* (%)^d^	613 (81.8)	415 (89.4)	97 (59.5)	101 (82.8)	<0.001
DHCA time (min), median (IQR)	8 (6-20)	7 (6-10)	20 (5-28)	27.5 (10-48)	<0.001
ECLS post-CPB, *n* (%)	9 (1.2)	5 (1.1)	0	4 (3.3)	0.058
Delayed sternal closure, *n* (%)	302 (40.3)	146 (31.5)	77 (47.2)	79 (64.8)	<0.001
ICU length of stay (days), median (IQR)	3 (2-5)	3 (2-5)	4 (2-6)	4 (3-7)	<0.001
Hospital length of stay (days), median (IQR)	9 (7-13)	9 (7-12)	9.5 (7-16)	11 (7-19.5)	0.001
30-Day mortality, *n* (%)	34 (4.5)	8 (1.7)	6 (3.7)	20 (16.4)	<0.001
90-Day mortality, *n* (%)	43 (5.7)	11 (2.4)	8 (4.9)	24 (19.7)	<0.001

a Small, haemodynamically insignificant, not closed.

b According to Leiden convention, with Yacoub classification in parentheses, where possible.

c Details of origin, course and branching patterns in Supplementary Material, Table S2.

d With use of single venous cannula (see Supplementary Material).

AXC: aortic cross-clamp; CPB: cardiopulmonary bypass; DHCA: deep hypothermic circulatory arrest; ECLS: extracorporeal life support; ICU: intensive care unit; IVS: intact ventricular septum; LAD: left anterior descending coronary artery; LIMA: left internal mammary artery; LVOTO: left ventricular outflow tract obstruction; PA: pulmonary artery; RVOTO: right ventricular outflow tract obstruction; TGA: transposition; VSD: ventricular septal defect.

### Operative

Balloon atrial septostomy was performed in 532 (71.0%) patients. Thirty-one (4.1%) patients underwent a surgical procedure prior to arterial switch, including isolated pulmonary artery banding in 13 (1.7%), aortic arch repair with banding in 12 (1.6%) and systemic-pulmonary artery shunt ± banding in 4 (0.5%), mostly performed elsewhere prior to referral in the early part of the series. Arterial switch was performed at a median age of 8 (IQR 5–16) days and beyond 28 days in 91 patients (12.1%).

Thirty-three (7.1%) patients in the TGA-IVS group had 1 or more small, haemodynamically insignificant VSDs identified on echo which were not closed, and often not found, during surgery. Three (2.5%) patients in the complex group had very large VSDs, which were not closed at arterial switch, rather treated with concomitant neopulmonary artery banding.

### Early outcomes

Nine (1.2%) infants required extracorporeal life support in the early postoperative period. The median length of stay on ICU was 3 days (IQR 2–5) and length of hospital stay was 9 days (IQR 7–13); both were longer in the complex group (*p* < 0.001).

There were 34 deaths within 30 days, with an overall early mortality of 4.5% [95% confidence interval (CI) 3.1–6.1]. Early mortality was higher in the complex group (20, 16.4%, *P* < 0.001) but similar between other groups: 8 (1.7%) in TGA-IVS and 6 (3.7%) in TGA-VSD (*P* = 0.21); there was no difference between those who underwent arterial switch before or after 28 days (*P* = 0.29). There were 12 intraoperative deaths but none since 1997. Early death was more common in the first 4 years (10/136, 7.4%) than in subsequent years (24/613, 3.9%) (Supplementary Material, Fig. S2) and has been low since 2000 (10/380, 2.6%), with only 1 death (1/215, 0.5%) amongst infants with non-complex TGA and the usual coronary pattern. A further 9 deaths occurred during the index admission due to concomitant conditions or complications of prolonged ICU stay, with an overall 90-day mortality of 5.7%.

**Figure 2:**
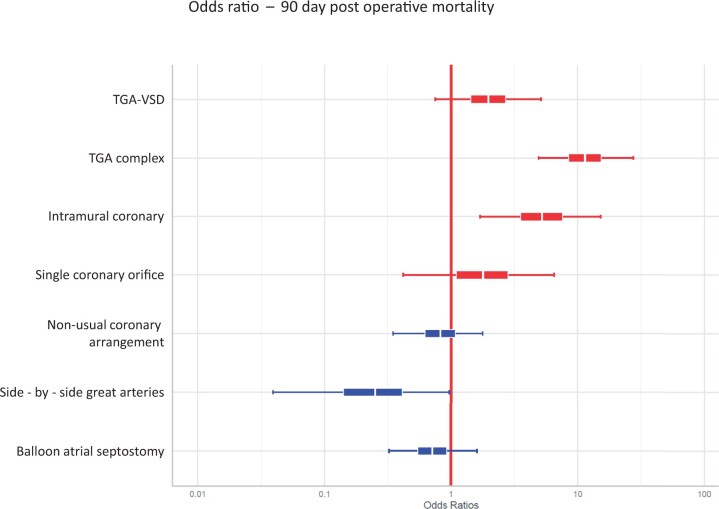
Forest plot of potential risk factors for 90-day mortality. The central line in each box is the mean estimate, the width of the box represents the central 50% and the whiskers corresponds to the 95% credible interval of the posterior distribution. TGA: transposition; VSD: ventricular septal defect.

Risk factors for 90-day mortality are shown in Table 2 and Fig. 2. Complex morphology [odds ratio (OR) 11.44, 95% CI 4.76–27.43] and intramural coronary artery (OR 5.17, 95% CI 1.61–15.91) were the most important factors, whilst for isolated VSD (OR 1.94, 95% CI 0.73–5.06), single coronary orifice (OR 1.75, 95% CI 0.43–6.56) and balloon atrial septostomy (OR 0.71, 95% CI 0.31–1.66), the compatibility intervals were wide so any association could not be excluded. Side-by-side great arteries were associated with reduced risk of death compared with aorta anterior to the pulmonary artery (OR 0.24, 95% CI 0.04–0.91). Overall, the model had a *C*-index of 0.85.

**Table 2:** Risk factors for 90-day mortality using a multivariable Bayesian analysis

Parameter	Odds ratio	Lower 2.5% CI	Upper 97.5% CI
TGA-IVS			
TGA-VSD	1.94	0.73	5.06
Complex TGA	11.44	4.76	27.43
Usual coronary arrangement, 1LCx-2R			
Non-usual coronary arrangement, other than 1LCx-2R	0.82	0.34	1.85
No intramural coronary artery			
Intramural coronary artery	5.17	1.61	15.91
More than 1 coronary orifice			
Single coronary orifice	1.75	0.43	6.56
Aorta anterior to PA			
Side-by-side great arteries	0.24	0.04	0.91
No balloon atrial septostomy			
Balloon atrial septostomy	0.71	0.31	1.66

Year of surgery and age at surgery were also included in the model (see Supplementary Material, Figs S2 and S3).

IVS: intact ventricular septum; PA: pulmonary artery; TGA: transposition; VSD: ventricular septal defect.

Early surgical reintervention was required in 32 (4.3%) patients (15 TGA-IVS, 3 TGA-VSD, 14 complex) and was more frequent in the complex group (*P* < 0.001) with no difference between the other groups (*P* = 0.59). These were most commonly coronary revision (6, 0.8%, including 3 intramurals), pulmonary artery repair (4, 0.5%), residual VSD closure (3, 0.4%), epicardial pacemaker implantation for heart block following VSD closure (3, 0.4%) or aortic arch repair (2, 0.3%). Emergency takedown was performed in 1 (0.1%) patient for deteriorating ventricular function not responding to conventional therapy. There were 2 (0.3%) transvenous pacemakers but no other early catheter reinterventions, as per our departmental policy.

Of the 3 patients with a large VSD who underwent neopulmonary artery banding, 1 died in the early postoperative period, 1 was subsequently septated and the other was deemed unseptatable, undergoing single-ventricle palliation and excluded from subsequent analysis.

### Late survival

Following discharge, during a median follow-up of 13.7 years (IQR 3.8–21.3), there were 15 late deaths (2.1% of hospital survivors) at a median of 4.0 years (IQR 1.5–15.0), 7 (1.5%) with TGA-IVS, 5 (3.1%) with TGA-VSD and 3 (2.5%) with complex TGA. Four patients died suddenly at home, 4 during or soon after reintervention, 2 from chronic heart failure (1 whilst awaiting transplantation, 1 from cardiac allograft vasculopathy), 1 from unrelated septicaemia and, in 4, the mode of death is unknown. All late deaths occurred in those with either the usual coronary pattern (1LCx-2R), circumflex from the right (1l-2CxR) or inverted circumflex/right (1RL-2Cx); none had an intramural, single orifice or interarterial course. The incidence of sudden unexpected death in hospital survivors was 0.04% per year of follow-up.

**Figure 3:**
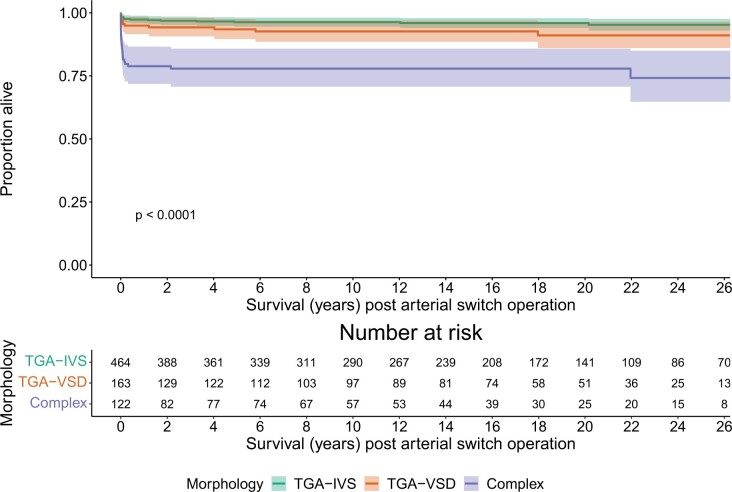
Kaplan–Meier curves showing survival following arterial switch, by morphological group.

Of 706 hospital survivors, late outcomes were available for 621 (88.0%). Overall survival was 93.8% (95% CI 92.1–95.6) at 1 year, 92.7% (95% CI 90.8–94.6) at 5 years, 92.5% (95% CI 90.6–94.5) at 10 years and 91.9% (95% CI 89.9–94.1) at 20 years. At latest follow-up, 446 (96.1%) with TGA-IVS, 150 (92.0%) with TGA-VSD and 96 (78.7%) with complex TGA were alive (Fig. 1). Survival by group is shown in Fig. 3 with additional data in Supplementary Material.

### Late surgical and catheter reinterventions

Following discharge, late surgical or catheter reinterventions were required in 118 (16.7%) survivors (Supplementary Material, Table S3). Eighty-two late surgical reoperations were performed in 66 (8.8%) patients (range 1–3), most often pulmonary artery patching (37 procedures in 34 patients, 4.5%). Neoaortic valve/root repair/replacement has been performed in 12 (1.6%) patients at a median of 13.2 (IQR 9.0–16.4) years. Late coronary reintervention was required in 4 (0.5%) patients (3 usual arrangement, 1 intramural [22]), 2 within the first year and the others at 10 and 26 years, either button reimplantation (3) or coronary artery bypass grafting (1). Late reoperation was more common in the complex group (25 procedures in 19 patients, 15.6%, *P* = 0.008) but similar in the TGA-IVS (39 in 32, 6.9%) and TGA-VSD (18 in 15, 9.2%) groups (*P* = 0.39).

One hundred and seventy-three late catheter interventions were performed in 87 (12.3%) survivors (range 1–8). Most reinterventions were ballooning/stenting of the branch pulmonary arteries (138 procedures in 68 patients, 9.1%), with ballooning of the neopulmonary valve in 12 (1.6%) and recoarctation in 6 (0.8%). Late catheter reintervention was more common in the complex group (50 procedures in 23 patients, 18.9%, *P* = 0.013) but similar in the TGA-IVS (94 in 47, 10.1%) and TGA-VSD (29 in 17, 10.4%) groups (*P* = 1.0).

**Figure 4:**
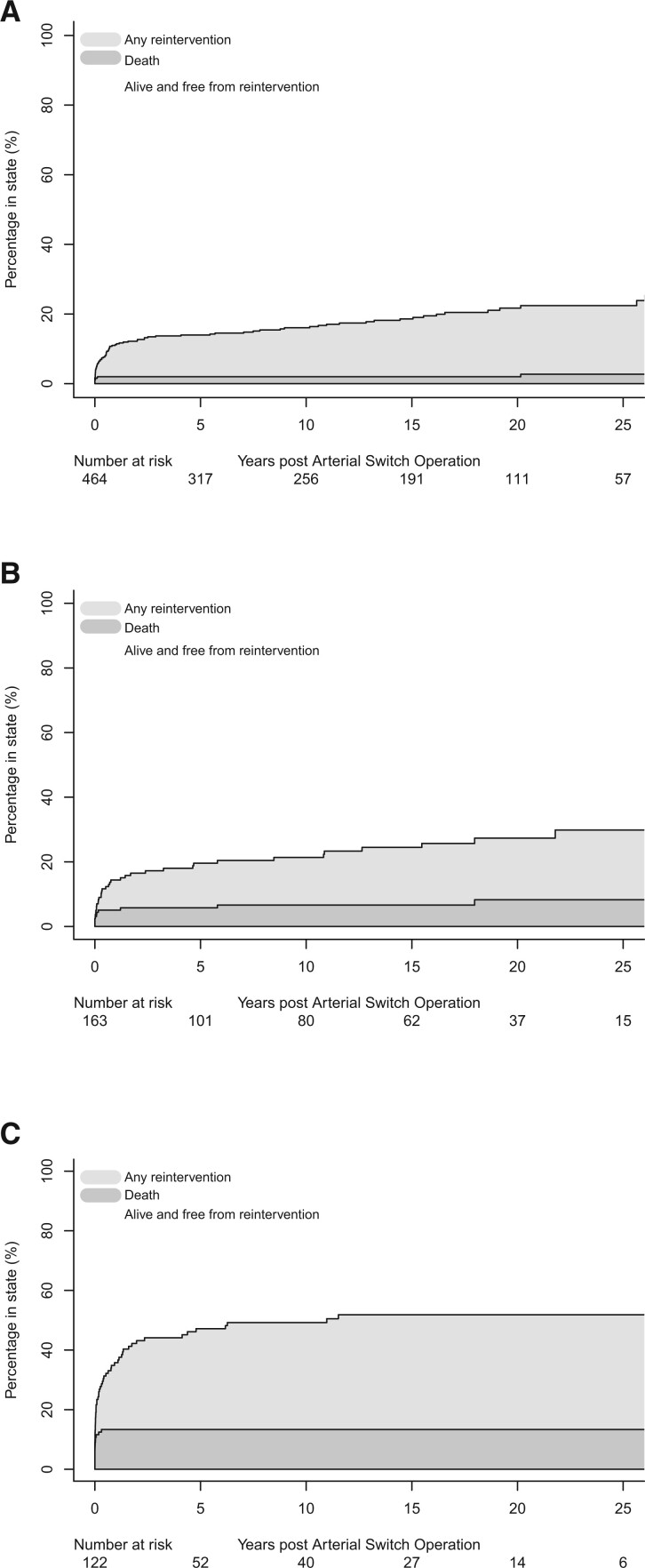
Cumulative incidence function plots for any reintervention, by morphological group: (**A**) transposition with intact ventricular septum, (**B**) transposition with ventricular septal defect and (**C**) complex transposition.

The cumulative incidence of surgical and/or catheter reintervention censored for death was 11.4% (95% CI 10.0–13.0) at 1 year, 16.0% (95% CI 14.5–17.5) at 5 years, 17.8% (95% CI 16.3–19.4) at 10 years and 22.7% (95% CI 21.0–24.0) at 20 years. The risks of reintervention by group are shown in Fig. 4 and the Supplementary Material.

## DISCUSSION

In this study, we demonstrate that the arterial switch can be performed using a reliable operative technique with consistently low early mortality, moderate rates of surgical and catheter reintervention and excellent late survival. Our overall early mortality of 2.6% since 2000 compares favourably with other contemporary series [7, 8, 14, 23]. However, patients in the complex group, with associated coarctation, aortic interruption, ventricular outflow tract obstruction or Taussig–Bing, and those with an intramural coronary artery were at greater risk of early death. Complex TGA was also associated with increased need for reintervention, but hospital survivors had similar long-term survival.

### Risk factors for early mortality

The most important risk factors for early death were complex morphology and intramural coronary artery; the latter remained associated with outcome despite a low event rate in the non-complex groups. It is uncertain whether patients with a single coronary orifice had increased risk of death as the compatibility interval was wide. Dealing with variations in coronary anatomy is the key technical challenge of the arterial switch whilst repair of associated lesions in the aortic arch or outflow tracts has greater impact on ischaemic and circulatory arrest times. In a meta-analysis of studies reporting patients undergoing arterial switch before 2000, Pasquali *et al.* [15] found that intramural and single ostium coronary patterns were associated with increased mortality. More recently, Metton *et al.* reported that intramural patterns remain associated with early mortality in the current era [24] whilst Fricke *et al.* identified left ventricular outflow tract obstruction, aortic arch obstruction and weight <2.5 kg as risk factors for early death, with no deaths amongst those with an intramural pattern [7]. Age at arterial switch and balloon atrial septostomy have also been identified as risk factors [25] but neither were apparent in our series. Unexpectedly, we found that side-by-side great arteries were associated with reduced risk of death, compared with aorta anterior to the pulmonary artery across all groups, but the reason for this is unclear.

A VSD has been identified to increase the risk of arterial switch, in both Risk Adjustment for Congenital Heart Surgery and Aristotle risk-adjustment models [12, 13]. Data from the European Congenital Heart Surgeons Association multi-institutional study identified VSD as the most important risk factor for early mortality [14] but may reflect the higher incidence of other associated lesions in this group, which we have classified as complex TGA. We found that an isolated VSD was less important than other factors in predicting the risk of early death.

### Reintervention and late outcomes

Early surgical reintervention was required in 4.3% of patients. Late catheter and/or surgical reinterventions were performed in 15.8% of patients, comparable with previous series [8, 9, 26]. In this and other series, the most common reason for late reintervention was branch pulmonary artery stenosis, reflecting a proactive approach to dealing with recurrent lesions. Both early and late reinterventions were more common in those with complex TGA but unlike some other reports [8, 26], an isolated VSD did not increase the risk of late reintervention.

We have previously reported on the fate of the neoaortic valve in the first decade of this cohort with a 97.7% freedom from aortic valve reoperation during childhood [27]. In the current series, neoaortic valve or root reoperation was performed in 1.6% of patients at a median of 13.2 years, which is similar to other large series: 2.7% at 13.3 years at the Mayo Clinic [9] and 4.0% at 14.5 years in Melbourne, increasing over time [7, 26]. Furthermore, early or late coronary reintervention was required in 10 (1.3%) patients, of whom 4 had an intramural and/or interarterial course. No deaths beyond 30 days occurred in those with an intramural, single orifice or interarterial coronary pattern. This low rate of late coronary-related complications following arterial switch using the trap-door technique [6], or pericardial hood [17] when required, has similarly been reported by the Melbourne group [26], in contrast to other large series [8]. The standardized trap-door technique therefore minimizes coronary reinterventions without an associated increase in neoaortic reoperations within the current extent of follow-up.

### Limitations

Our findings are subject to the limitations inherent to retrospective cohort studies. Detailed descriptions of morphology were obtained from contemporaneous operation notes, without reference to echocardiographic or angiographic studies. We only included patients deemed suitable for arterial switch and therefore patients with more complex patterns were excluded. Data are limited to a single, high-volume institution with a consistent operative technique used by all surgeons throughout the study period which may limit its generalizability. Our low event rate for early mortality also limited the assessment of potential risk factors. In the early years, many patients with complex anatomy were referred from elsewhere in the UK and Europe, often following an initial palliative procedure, which may have contributed to a higher throughput and complexity in this period (see Supplementary Material).

## CONCLUSION

The arterial switch has revolutionized the natural history of TGA. We demonstrate that it can be performed with low early mortality, moderate rates of reintervention and excellent long-term survival, using a standardized institutional technique throughout the series which we have previously shown to be reproducible and suitable for mentoring to avoid a learning curve [11]. Complex TGA with concomitant lesions and an intramural coronary artery were independently associated with increased early mortality but with a low event rate, an isolated VSD and other coronary patterns had less impact on survival. As this cohort enters their fourth decade, the very late outcomes of arterial switch including the fate of the neoaortic root, are not yet known. These patients require long-term follow-up to determine the ongoing burden of disease and need for reintervention during adulthood.

## Supplementary Material

ivad070_Supplementary_Data

## Data Availability

The data underlying this article cannot be shared publicly to maintain the confidentiality of the individuals involved. The anonymized data may be shared on reasonable request to the corresponding author. **Nigel E. Drury:** Conceptualization; Data curation; Formal analysis; Investigation; Methodology; Project administration; Validation; Writing—original draft. **Shafi Mussa:** Conceptualization; Investigation; Writing—review & editing. **John Stickley:** Data curation; Formal analysis; Methodology; Software; Visualization; Writing—review & editing. **Oliver Stumper:** Resources; Writing—review & editing. **Adrian Crucean:** Validation; Writing—review & editing. **Rami Dhillon:** Resources; Writing—review & editing. **Anna N. Seale:** Resources; Writing—review & editing. **Phil Botha:** Resources; Writing—review & editing. **Natasha E. Khan:** Resources; Writing—review & editing. **David J. Barron:** Resources; Supervision; Writing—review & editing. **William J. Brawn:** Resources; Writing—review & editing. **Timothy J. Jones:** Conceptualization; Data curation; Investigation; Resources; Supervision; Writing—review & editing. Interdisciplinary CardioVascular and Thoracic Surgery thanks Emre Belli, Julie Cleuziou and the other anonymous reviewer(s) for their contribution to the peer review process of this article.

## ABBREVIATIONS

CI: Confidence interval
ICU: Intensive care unit
IQR: Interquartile range
IVS: Intact ventricular septum
OR: Odds ratio
TGA: Transposition
VSD: Ventricular septal defect

## References

[1] Samánek M. Congenital heart malformations: prevalence, severity, survival, and quality of life. Cardiol Young 2000;10:179–85. 10824896 10.1017/s1047951100009082

[2] Liebman J , Cullum L, Belloc NB. Natural history of transposition of the great arteries: anatomy and birth and death characteristics. Circulation 1969;40:237–62. 4240356 10.1161/01.cir.40.2.237

[3] Jatene AD , Fontes VF, Paulista PP, de Souza LC, Neger F, Galantier M et al Successful anatomic correction of transposition of the great vessels. Arq Bras Cardiol 1975;28:461–4. 1200893

[4] Villafañe J , Lantin-Hermoso MR, Bhatt AB, Tweddell JS, Geva T, Nathan M et al; American College of Cardiology’s Adult Congenital and Pediatric Cardiology Council. D-transposition of the great arteries: the current era of the arterial switch operation. J Am Coll Cardiol 2014;64:498–511. 25082585 10.1016/j.jacc.2014.06.1150 PMC4340094

[5] Lecompte Y , Zannini L, Hazan E, Jarreau MM, Bex JP, Tu TV et al Anatomic correction of transposition of the great arteries. J Thorac Cardiovasc Surg 1981;82:629–31. 7278356

[6] Brawn WJ , Mee RB. Early results for anatomic correction of transposition of the great arteries and for double-outlet right ventricle with subpulmonary ventricular septal defect. J Thorac Cardiovasc Surg 1988;95:230–8. 3339890

[7] Fricke TA , d'Udekem Y, Richardson M, Thuys C, Dronavalli M, Ramsay JM et al Outcomes of the arterial switch operation for transposition of the great arteries: 25 years of experience. Ann Thorac Surg 2012;94:139–45. 22607787 10.1016/j.athoracsur.2012.03.019

[8] Khairy P , Clair M, Fernandes SM, Blume ED, Powell AJ, Newburger JW et al Cardiovascular outcomes after the arterial switch operation for D-transposition of the great arteries. Circulation 2013;127:331–9. 23239839 10.1161/CIRCULATIONAHA.112.135046

[9] Raju V , Burkhart HM, Durham LA, Eidem BW, Phillips SD, Li Z et al Reoperation after arterial switch: a 27-year experience. Ann Thorac Surg 2013;95:2105–13. 23618522 10.1016/j.athoracsur.2013.02.040

[10] Tobler D , Williams WG, Jegatheeswaran A, Van Arsdell GS, McCrindle BW, Greutmann M et al Cardiac outcomes in young adult survivors of the arterial switch operation for transposition of the great arteries. J Am Coll Cardiol 2010;56:58–64. 20620718 10.1016/j.jacc.2010.03.031

[11] Mussa S , Drury NE, Stickley J, Khan NE, Jones TJ, Barron DJ et al Mentoring new surgeons: can we avoid the learning curve? Eur J Cardiothorac Surg 2017;51:291–9. 28186266 10.1093/ejcts/ezw293

[12] Jenkins KJ , Gauvreau K, Newburger JW, Spray TL, Moller JH, Iezzoni LI et al Consensus-based method for risk adjustment for surgery for congenital heart disease. J Thorac Cardiovasc Surg 2002;123:110–8. 11782764 10.1067/mtc.2002.119064

[13] O'Brien SM , Jacobs JP, Clarke DR, Maruszewski B, Jacobs ML, Walters HL et al Accuracy of the aristotle basic complexity score for classifying the mortality and morbidity potential of congenital heart surgery operations. Ann Thorac Surg 2007;84:2027–37. 18036930 10.1016/j.athoracsur.2007.06.031

[14] Sarris GE , Chatzis AC, Giannopoulos NM, Kirvassilis G, Berggren H, Hazekamp M et al; European Congenital Heart Surgeons Association. The arterial switch operation in Europe for transposition of the great arteries: a multi-institutional study from the European Congenital Heart Surgeons Association. J Thorac Cardiovasc Surg 2006;132:633–9. 16935120 10.1016/j.jtcvs.2006.01.065

[15] Pasquali SK , Hasselblad V, Li JS, Kong DF, Sanders SP. Coronary artery pattern and outcome of arterial switch operation for transposition of the great arteries: a meta-analysis. Circulation 2002;106:2575–80. 12427654 10.1161/01.cir.0000036745.19310.bb

[16] Kang N , de Leval MR, Elliott M, Tsang V, Kocyildirim E, Sehic I et al Extending the boundaries of the primary arterial switch operation in patients with transposition of the great arteries and intact ventricular septum. Circulation 2004;110:II123–7. 15364850 10.1161/01.CIR.0000138221.68312.33

[17] Parry AJ , Thurm M, Hanley FL. The use of ‘pericardial hoods’ for maintaining exact coronary artery geometry in the arterial switch operation with complex coronary anatomy. Eur J Cardiothorac Surg 1999;15:159–64. 10219548 10.1016/s1010-7940(98)00314-5

[18] Gittenberger-de Groot AC , Koenraadt WMC, Bartelings MM, Bökenkamp R, DeRuiter MC, Hazekamp MG et al Coding of coronary arterial origin and branching in congenital heart disease: the modified Leiden Convention. J Thorac Cardiovasc Surg 2018;156:2260–9. 30243713 10.1016/j.jtcvs.2018.08.009

[19] Massoudy P , Baltalarli A, de Leval MR, Cook A, Neudorf U, Derrick G et al Anatomic variability in coronary arterial distribution with regard to the arterial switch procedure. Circulation 2002;106:1980–4. 12370223 10.1161/01.cir.0000033518.61709.56

[20] Yacoub MH , Radley-Smith R. Anatomy of the coronary arteries in transposition of the great arteries and methods for their transfer in anatomical correction. Thorax 1978;33:418–24. 694795 10.1136/thx.33.4.418 PMC470907

[21] Buerkner P-C. brms: an R package for Bayesian multilevel models using Stan. J Stat Software 2017;80:1–28.

[22] Stoll VM , Hudsmith LE, Drury NE, Barron DJ. Late complication of intramural coronary transfer during the arterial switch operation. Interact CardioVasc Thorac Surg 2019;28:638–9. 30380068 10.1093/icvts/ivy289

[23] Moll M , Michalak KW, Sobczak-Budlewska K, Moll JA, Kopala M, Szymczyk K et al Coronary artery anomalies in patients with transposition of the great arteries and their impact on postoperative outcomes. Ann Thorac Surg 2017;104:1620–8. 28648541 10.1016/j.athoracsur.2017.03.078

[24] Metton O , Calvaruso D, Gaudin R, Mussa S, Raisky O, Bonnet D et al Intramural coronary arteries and outcome of neonatal arterial switch operation. Eur J Cardiothorac Surg 2010;37:1246–53. 20153213 10.1016/j.ejcts.2009.12.042

[25] O'Byrne ML , Glatz AC, Song L, Griffis HM, Millenson ME, Gillespie MJ et al Association between variation in preoperative care before arterial switch operation and outcomes in patients with transposition of the great arteries. Circulation 2018;138:2119–29. 30474422 10.1161/CIRCULATIONAHA.118.036145 PMC6261315

[26] Fricke TA , Buratto E, Weintraub RG, Bullock A, Wheaton G, Grigg L et al Long-term outcomes of the arterial switch operation. J Thorac Cardiovasc Surg 2022;163:212–9. 33715839 10.1016/j.jtcvs.2021.01.134

[27] Lo Rito M , Fittipaldi M, Haththotuwa R, Jones TJ, Khan N, Clift P et al Long-term fate of the aortic valve after an arterial switch operation. J Thorac Cardiovasc Surg 2015;149:1089–94. 25543959 10.1016/j.jtcvs.2014.11.075

