# Supplementary material for: Outcomes of the arterial switch for transposition during infancy using a standardized approach over 30 years
Source: Interdiscip Cardiovasc Thorac Surg. 2023 May 10;37(1):ivad070. doi: 10.1093/icvts/ivad070 (PMC10348837; doi:10.1093/icvts/ivad070)
Supplement: ivad070_Supplementary_Data [file ivad070_supplementary_data.pdf]

SUPPLEMENTARY MATERIALS

Expanded Methods

All patients undergoing arterial switch during infancy at Birmingham Children's Hospital, UK between January 1988 and December 2018 were included. Patients over one year of age at arterial switch were excluded as they were predominately referred early in the series, mostly following banding and/or shunt formation elsewhere and are not representative of our current practice of neonatal anatomical repair.

Preoperative diagnosis and assessment were made by echocardiography and ductal patency was maintained with intravenous prostaglandin as required. Patients with inadequate mixing underwent emergency balloon atrial septostomy prior to repair. Initial palliative procedures were performed to relieve coarctation, increase or restrict pulmonary blood flow, or train the subpulmonary ventricle, as indicated. Arterial switch was used in all patients who were deemed suitable for anatomical correction and was not influenced by preoperative assessment of coronary anatomy. Additional procedures were performed at the time of arterial switch to treat residual associated lesions.

Operative technique

The surgical technique for arterial switch at our institution remained remarkably consistent throughout the study period. Under general anaesthesia and following median sternotomy, an autologous pericardial patch was harvested, and the proximal great vessels mobilized. Bicaval cannulation was used with cooling to a nasopharyngeal temperature of 28-32°C, although in the early years of this series, a single right atrial cannula was used in infants <3kg or without a VSD, with cooling to 22°C and a brief period of circulatory arrest for closure of the atrial communication. Alphastat pH strategy was employed during cardiopulmonary bypass (CPB). The arterial duct was double ligated, divided and both ends oversewn. Further mobilization of the pulmonary arteries to the hilar vessels was performed. The ascending aorta was clamped and cold crystalloid cardioplegia administered via the aortic root.

Additional procedures were performed to correct concomitant lesions, as required. Transatrial approach to a VSD was used routinely and patched with a bovine pericardium or double velour Dacron patch and interrupted pledgetted polypropylene sutures. The ventricular outflow tracts were carefully inspected and, when necessary, resection performed to ensure unobstructed blood flow. Any aortic repair was performed under deep hypothermic circulatory arrest at 18°C or selective antegrade cerebral perfusion with the arterial cannula advanced into the brachiocephalic artery. Isolated coarctation was treated by resection and extended end-to-end anastomosis, whilst aortic arch hypoplasia underwent resection of the coarctation and either extended end-to-end anastomosis or homograft patch augmentation of the underside of the arch. Following arch repair, the vascular clamp and arterial cannula were repositioned enabling re-perfusion of the aortic arch and descending aorta.

The ascending aorta was then transected 3-5mm above the aortic valve commissures and the coronary arteries were excised with a generous cuff of aortic sinus tissue and mobilized. The resultant aortic defects were repaired with untreated autologous pericardium as a single patch. The main pulmonary artery was transected at the same level as the aorta and the coronary artery buttons were relocated to medially hinged trapdoor incisions to construct the proximal neoaorta. Intramural coronary arteries were mobilized with generous cuffs of aortic wall, taking

down the valve commissure or laying opening the ostia as required. If unable to transfer as described above, a homograft tissue patch was used to augment the receiving aortic sinus, incorporating the intramural cuff with minimal mobilization. The Lecompte manoeuvre was performed whenever possible; in those with side-by-side great vessels, the pulmonary artery bifurcation was relocated as necessary by incising the branch pulmonary artery to accommodate the main pulmonary artery, oversewing the native opening. The neoaorta was reconstructed, the heart re-perfused and reconstruction of the neopulmonary artery was completed while rewarming and with the heart beating.

CPB was discontinued with inotropic support and routine left atrial pressure line monitoring. Primary sternal closure was undertaken whenever feasible, or on the ICU as a delayed procedure, usually within 24-48 hours. The use of delayed sternal closure was determined by the degree of cardiac swelling and ventricular function post-CPB, along with the cumulative ischaemic time.

Data collection

Detailed morphological data were collected from the operative records. Coronary arteries were classified as intramural if one or more passed obliquely through the aortic wall. Single coronary orifice was defined as the left anterior descending, circumflex and right coronary arteries arising from a common origin in the ascending aorta, discounting minor accessory arteries.

Ethnicity was obtained from routine UK National Health Service data collection, collapsed to the five groups used by the National Institute for Cardiovascular Outcomes Research (NICOR). For missing data, the most likely ethnicity was derived by assessing the ethnic group most associated with the patient's surname in our hospital database. Where this data was missing, it was imputed based on the patient's surname from a regional database of almost 60,000 surnames with confirmed ethnicity. Data on late deaths were obtained from the UK Office for National Statistics (ONS) using the Demographics Batch Service Bureau (DBSB) service. Date of latest follow-up was the last documented clinic review by the patient's cardiologist or death. Surgical and catheter reinterventions were defined as any cardiovascular surgery or procedure performed at cardiac catheterisation, respectively, following completion of arterial switch.

Statistical analysis

Event rates for reinterventions were estimated using cumulative incidence function [S1] with death as the competing risk [S2]. To estimate risk factors for all-cause mortality, a logistic regression model was developed using a Bayesian method in the R BRMS package [S3], with 90-day all-cause mortality as the outcome. Variables were selected based on expert opinion and previous studies alongside the requirement for data availability because of the duration of patient selection. The following variables were included in the model: year of surgery, age at surgery, morphological group, non-usual coronary pattern, intramural coronary, single coronary orifice, aorta-pulmonary artery arrangement, and balloon atrial septostomy. A number of prior distributions were assessed. The published model used a sceptical prior with a Student t distribution, with mean zero and 3 degrees of freedom. Checks were made for co-linearity of variables. The model's development diagnostics were reviewed using summary statistics and graphical output to ensure that the model had converged. Model performance was assessed using leave-one-out cross-validation. 95% compatibility intervals were derived from the posterior distribution and are presented.

Expanded Results

Since 1988, arterial switch has been our procedure of choice for the anatomical correction of transposition and other operations have only been performed when this approach was deemed unsuitable: 62 patients with transposition, VSD and left ventricular outflow tract obstruction underwent Rastelli operation (49), REV (Réparation à l'Etage Ventriculaire) procedure (12) or Nikaidoh procedure (1); 2 patients with transposition, VSD and subaortic stenosis underwent Damus-Rastelli procedure; and 96 patients with complex transposition, including with double inlet left ventricle, ventricular hypoplasia, tricuspid atresia, atrial isomerism, unbalanced atrioventricular septal defect or straddling atrioventricular valve, who were deemed unsuitable for biventricular repair underwent univentricular palliation. No isolated Mustard or Senning procedures for transposition or Kawashima intraventricular repair for Taussig-Bing were performed during this period. In addition, arterial switch was performed in 10 patients who underwent Mustard (5) or Senning (5) takedown and in 91 patients as part of a double switch for congenitally corrected transposition who are not included in this analysis.

The number of infants undergoing arterial switch at our institution per year by morphological group is depicted in Supplementary Figure 1. Variations in early mortality over time and by age are shown as splines in Supplementary Figures 2 and 3. Complete demographic data was available for all patients, except for ethnicity which was confirmed in 738 (98.5%) patients with imputation required for the other 11 patients, as shown in Supplementary Table 1. 168 (22.4%) patients were non-electively ventilated prior to arterial switch and one (0.1%) was referred from another institution on extra-corporeal life support (ECLS).

Morphology

Of 122 patients with complex transposition, 109 (89.3%) had a significant interventricular communication, 86 (70.5%) had coarctation of the aorta, 32 (26.2%) had ventricular outflow tract obstruction, and 10 (8.2%) had interrupted aortic arch (eight type A, two type B).

Detailed coronary artery patterns and associated mortality following arterial switch are provided in Supplementary Table 2. In the 36 patients with an intramural pattern, the left coronary artery was intramural in 19 (53%), the left anterior descending artery in 8 (22%), the right coronary artery in 6 (17%), and both the left and right coronary arteries in 3 (8%). An intramural course was uncommon in patients with usual sinuses of origin (5, 1.0%) but was frequently associated with an interarterial course in those originating from sinus 2 only (24, 52.2%). In 26 (3.5%) patients, the anatomy could not be categorised according to the Yacoub classification

All deaths in those with coronary patterns other than the usual arrangement (1LCx-2R), circumflex from the right (1L-2CxR), and inverted circumflex/right (1RL-2Cx), occurred within 30 days of surgery. Both patients with the combination of a single coronary orifice and an intramural pattern died. The origin, course and branching pattern of coronary arteries originating only from sinus 2 is highly variable due to the greater number of possible arrangements, and the variants observed in this series are shown in Supplementary Figure 4.

Early outcomes

Nine (1.2%) patients required ECLS in the early postoperative period for poor ventricular function. Four failed to wean from CPB: three with complex transposition (two with Taussig-Bing, one with mesocardia and arch hypoplasia), and one with TGA-IVS and an intramural/interarterial left coronary artery. Five patients developed low cardiac output on ICU

and required ECLS, four of whom underwent emergency reintervention for coronary revision: three with TGA-IVS (one intramural) and one with complex transposition. Of these nine patients, five were successfully weaned from ECLS and remain alive, two were weaned but died (one during the index admission, one whilst awaiting heart transplant), and two were unable to be weaned and care was withdrawn.

Survival by group

Of 706 hospital survivors, 504 remained under local follow-up, 189 at other UK centres, and 13 abroad; late outcomes were available for 621 (88.0%).

Survival in the TGA-IVS group was 97.3% (95% CI 95.9-98.8) at 1 year, 96.3% (95% CI 94.5-98.1) at 5 years, 96.3% (95% CI 94.5-98.1) at 10 years, 95.9% (95% CI 94.0-97.9) at 15 years, and 95.9% (95% CI 94.0-97.9) at 20 years.

Survival in the TGA-VSD group was 94.9% (95% CI 91.6-98.4) at 1 year, 93.5% (95% CI 89.6-97.5) at 5 years, 92.6% (95% CI 88.-97.0) at 10 years, 92.6% (95% CI 88.5-97.0) at 15 years, and 91.1% (95% CI 86.1-96.4) at 20 years.

Survival in the complex transposition group was 78.8% (95% CI 71.8-86.6) at 1 year, and 77.9% (95% CI 70.7-85.8) at 5, 10, 15 and 20 years.

Incidence of reintervention

The cumulative incidence of surgical reintervention censored for death was 8.5% (95% CI 7.1-10.1) at 1 year, 11.3% (95% CI 9.8-13.0) at 5 years, 12.1% (95% CI 10.5-13.7) at 10 years, and 14.5% (95% CI 12.7-16.4) at 20 years. The cumulative incidence of catheter reintervention censored for death was 6.9% (95% CI 5.3-8.9) at 1 year, 10.4% (95% CI 8.6-12.3) at 5 years, 11.7% (95% CI 9.9-13.7) at 10 years, and 14.8% (95% CI 13.0-16.7) at 20 years.

Cumulative incidence of reintervention following arterial switch by intervention type is shown in Supplementary Figure 5, and by morphological group in Supplementary Figures 6-7

The cumulative incidence of any reintervention censored for death in the TGA-IVS group was 9.2% (95% CI 8.0-10.6) at 1 year, 12.0% (95% CI 10.7-13.3) at 5 years, 14.1% (95% CI 12.8-15.4) at 10 years, and 19.7% (95% CI 18.5-21.0) at 20 years. The cumulative incidence of any reintervention censored for death in the TGA-VSD group was 9.3% (95% CI 6.2-13.1) at 1 year, 13.8% (95% CI 10.4-17.7) at 5 years, 14.7% (95% CI 11.0-19.0) at 10 years, and 19.1% (95% CI 15.3-23.2) at 20 years. The cumulative incidence of any reintervention censored for death in the complex transposition group was 22.4% (95% CI 16.6-28.8) at 1 year, 33.8% (95% CI 27.8-40.0) at 5 years, 35.8% (95% CI 29.8-42.0) at 10 years, and 38.5% (95% CI 32.4-44.6) at 20 years.

Supplementary References

S1. Fine JP, Gray RJ. A proportional hazards model for the subdistribution of a competing risk. *J Am Stat Assoc* 1999;94:496-509.

S2. Austin PC, Lee DS, Fine JP. Introduction to the analysis of survival data in the presence of competing risks. *Circulation* 2016;133:601-609.

S3. Buerkner P-C. brms: An R package for Bayesian multilevel models using Stan. *J Stat Software* 2017;80:1-28.

SUPPLEMENTARY TABLES

Supplementary Table 1. Additional patient characteristics, by morphological group.

Characteristic	**Overall** n=749	**TGA-IVS** n=464 (61.9%)	**TGA-VSD** n=163 (21.8%)	**Complex TGA** n=122 (16.3%)	p value
Ethnic origin, n (%)					0.72
White	599 (80.0)	375 (80.8)	128 (78.5)	96 (78.7)	
Asian	100 (13.4)	62 (13.4)	21 (12.9)	17 (13.9)	
Black	11 (1.5)	4 (0.9)	4 (2.5)	3 (2.5)	
Chinese	4 (0.5)	3 (0.6)	0	1 (0.8)	
Mixed/other	35 (4.7)	20 (4.3)	10 (6.1)	5 (4.1)	
Associated anomalies, n (%)					
Bicuspid neoaortic valve	22 (2.9)	11 (2.4)	8 (4.9)	3 (2.5)	0.24
Dextrocardia	5 (0.7)	4 (0.9)	0	1 (0.8)	0.44
Straddling mitral or tricuspid valve	1 (0.1)	0	0	1 (0.8)	0.08
Atrioventricular septal defect	2 (0.3)	0	0	2 (1.6)	0.006
Intervention prior to ASO, n (%)					
Balloon atrial septostomy	532 (71.0)	395 (85.1)	99 (60.7)	38 (31.1)	<0.001
Isolated PA banding	13 (1.7)	6 (1.3)	5 (3.1)	2 (1.6)	0.57
Aortic arch repair + PA banding	12 (1.6)	0	0	12 (9.8)	<0.001
Blalock-Taussig or central shunt	4 (0.5)	2 (0.4)	0	2 (1.6)	0.15
Other intervention	2 (0.3)	1 (0.2)	1 (0.6)	0	0.53
Pre-op non-elective ventilation, n (%)	168 (22.4)	110 (23.7)	24 (14.7)	34 (27.9)	0.02

ASO, arterial switch operation; PA, pulmonary artery.

Supplementary Table 2. Detailed coronary artery patterns and associated mortality following arterial switch.

Coronary artery origins, n (%)	**Frequency** n=749	**30-day mortality**	**90-day mortality**
1LCx-2R	514 (68.6)	19 (3.7)	26 (5.1)
Usual position in sinuses (A)	507 (67.7)	16 (3.2)	23 (4.5)
Adjacent to commissure (C)	7 (0.9)	3 (42.9)	3 (42.9)
1L-2CxR (D)	108 (14.4)	4 (3.7)	5 (4.6)
1RL-2Cx (E)	60 (8.0)	3 (5.0)	4 (5.0)
1R-2LCx (E)	4 (0.5)	0	0
1Cx-2LR	0	-	-
1RCx-2L	0	-	-
Originating from sinus 1 only	17 (2.3)	3 (17.6)	3 (17.6)
1RLCx *	15 (2.0)	3 (20.0)	3 (20.0)
1R,1LCx	2 (0.3)	0	0
Originating from sinus 2 only	46 (6.1)	5 (10.9)	5 (20.9)
2LCxR retropulmonary LCA *	13 (1.7)	1 (7.7)	1 (7.7)
2RLCx anteroaortic LCA *	2 (0.3)	0	0
2RLCx retropulmonary common trunk *	1 (0.1)	0	0
2CxRL retropulmonary Cx, anteroaortic LAD *	1 (0.1)	0	0
2LCxR unknown course *	5 (0.7)	0	0
2LCx,2R interarterial LCA	19 (2.5)	4 (21.1)	4 (21.1)
2L,2CxR interarterial LAD, retropulmonary Cx	5 (0.7)	0	0
Intramural coronary origin(s)	36 (4.8)	7 (19.4)	7 (19.4)
2LCx,2R / 2L,2CxR	24 (3.2)	4 (16.7)	4 (16.7)
1LCx-2R	5 (0.7)	1 (20.0)	1 (20.0)
1L-2CxR	4 (0.5)	0	0
1RLCx *	2 (0.3)	2 (100)	2 (100)
1RL-2Cx	1 (0.1)	0	0
Single coronary orifice (B)	37 (4.9)	4 (10.8)	4 (10.8)

Sinus origins according to the Leiden convention, with Yacoub classification in parentheses, where possible. Cx, circumflex coronary artery; LAD, left anterior descending coronary artery; LCA, left coronary artery.

* Contributes to single coronary orifice (B) count.

Supplementary Table 3. Late surgical and catheter reinterventions after arterial switch, by morphological group (n=749).

Late reinterventions	**Overall** n=749	**TGA-IVS** n=464 (61.7%)	**TGA-VSD** n=163 (21.8%)	**Complex TGA** n=122 (16.3%)
**Required surgical reoperation(s), n (%)**	66 (8.8)	32 (6.9)	15 (9.2)	19 (15.6)
Pulmonary arterioplasty, n (%)	37 in 34 (4.5)	17 in 15 (3.2)	8 (4.9)	12 in 11 (9.0)
RVOT resection/patching, n (%)	10 in 8 (1.1)	4 in 3 (0.6)	1 (0.6)	5 in 4 (3.3)
RV-PA conduit insertion, n (%)	4 (0.5)	1 (0.2)	2 (1.2)	1 (0.8)
RV-PA conduit change, n (%)	5 in 3 (0.4)	2 in 1 (0.2)	1 (0.6)	2 in 1 (0.8)
Neopulmonary valve replacement, n (%)	1 (0.1)	0	0	1 (0.8)
Neoaortic valve/root, n (%)	12 (1.6)	8 (1.7)	2 (1.2)	2 (1.6)
Neoaortic valve replacement, n (%)	6 (0.8)	3 (0.6)	1 (0.6)	2 (1.6)
Neoaortic valve repair, n (%)	1 (0.1)	1 (0.2)	0	0
Bentall procedure, n (%)	4 (0.5)	3 (0.6)	1 (0.6)	0
Valve-sparing root replacement, n (%)	1 (0.1)	1 (0.2)	0	0
Aortic ascending/arch/coarctation repair, n (%)	3 (0.4)	2 (0.4)	0	1 (0.8)
Coronary intervention, n (%)	4 (0.5)	3 (0.6)	1 (0.6)	0
Mitral valve repair/replacement, n (%)	7 in 5 (0.7)	2 (0.4)	1 (0.6)	4 in 2 (1.6)
Tricuspid valve repair, n (%)	2 (0.3)	0	0	2 (1.6)
LVOT resection, n (%)	3 (0.4)	1 (0.2)	1 (0.6)	1 (0.8)
VSD closure, n (%)	2 (0.3)	0	0	2 (1.6)
Pulmonary vein repair, n (%)	2 in 1 (0.1)	2 in 1 (0.2)	0	0
Superior CP shunt/Fontan procedure, n (%)	2 in 1 (0.1)	0	0	2 in 1 (0.8)
LVAD implant & explant, n (%)	1 (0.1)	1 (0.2)	0	0
Heart transplantation, n (%)	1 (0.1)	0	1 (0.6)	0
Epicardial permanent pacemaker, n (%)	2 (0.3)	0	0	2 (1.6)
Other, n (%)	2 (0.3)	0	1 (0.6)	1 (0.8)
Total surgical reoperations, n	82	39	18	25
**Required catheter reintervention(s), n (%)**	87 (11.6)	47 (10.1)	17 (10.4)	23 (18.9)
Pulmonary artery balloon, n (%)	121 in 63 (8.4)	76 in 42 (9.1)	19 in 13 (8.0)	26 in 10 (8.2)
Pulmonary artery stent, n (%)	17 in 15 (2.0)	8 in 7 (1.5)	5 in 4 (2.5)	4 (3.3)
Neopulmonary valve balloon, n (%)	14 in 12 (1.6)	3 (0.6)	4 (2.5)	7 in 5 (4.1)
RVOT balloon, n (%)	1 (0.1)	0	0	1 (0.8)
Recoarctation balloon, n (%)	6 (0.8)	1 (0.2)	0	5 (4.1)
Superior vena cava balloon, n(%)	1 (0.1)	0	0	1 (0.8)
Pulmonary vein balloon, n (%)	1 (0.1)	1 (0.2)	0	0
RV-PA conduit balloon, n (%)	1 (0.1)	1 (0.2)	0	0
VSD device closure, n (%)	2 (0.3)	0	1 (0.6)	1 (0.8)
ASD device closure, n (%)	2 (0.3)	2 (0.4)	0	0
Atrial septal stent, n (%)	1 (0.1)	1 (0.2)	0	0
Pulmonary artery occlusion device, n (%)	1 (0.1)	0	0	1 (0.8)
Atrial arrhythmia ablation, n (%)	2 (0.3)	1 (0.2)	0	1 (0.8)

Transvenous permanent pacemaker, n (%)	1 (0.1)	0	0	1 (0.8)
Other, n (%)	2 in 1 (0.1)	0	0	2 in 1 (0.8)
Total catheter reinterventions, n	173	94	29	50

ASD, atrial septal defect; CP, cavopulmonary; IVS, intact ventricular septum; LVAD, left ventricular assist device; LVOT, left ventricular outflow tract; RVOT, right ventricular outflow tract; RV-PA, right ventricle to pulmonary artery; TGA, transposition of the great arteries; VSD, ventricular septal defect.

SUPPLEMENTARY FIGURES

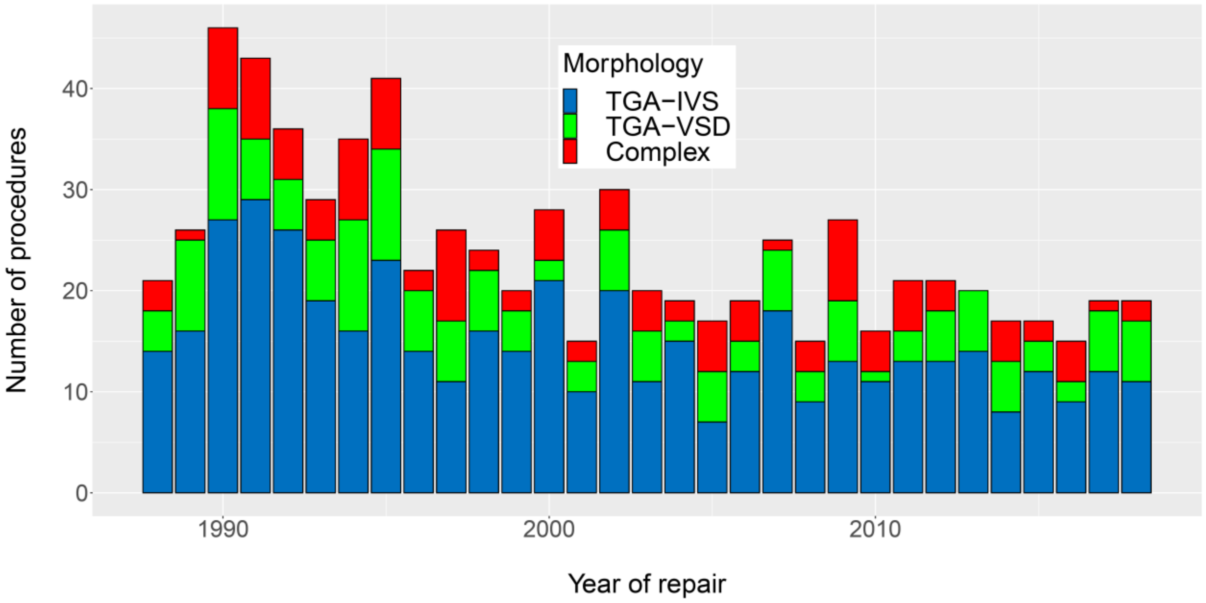

Supplementary Figure 1. Histogram of the number of infants undergoing arterial switch at our institution per year by morphological group.

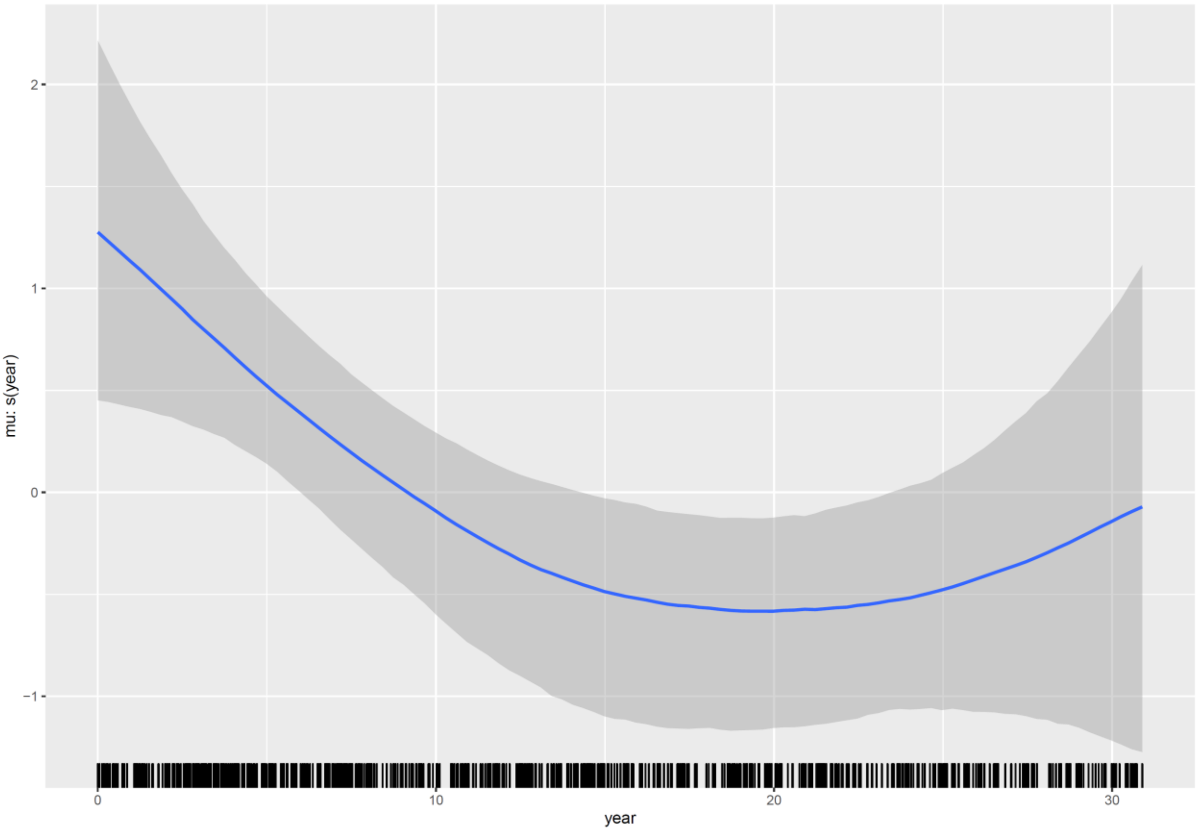

Supplementary Figure 2. Spline showing variation in early mortality over time.

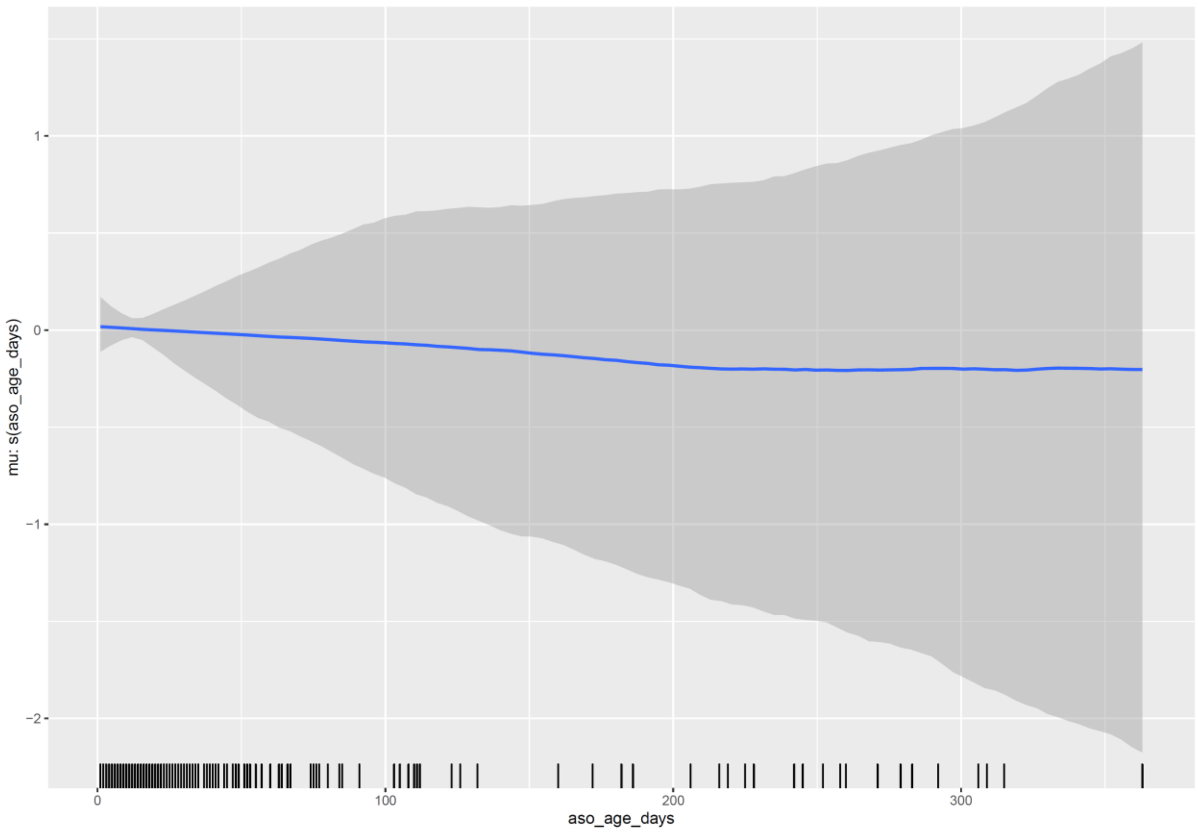

Supplementary Figure 3. Spline showing variation in early mortality by age at arterial switch.

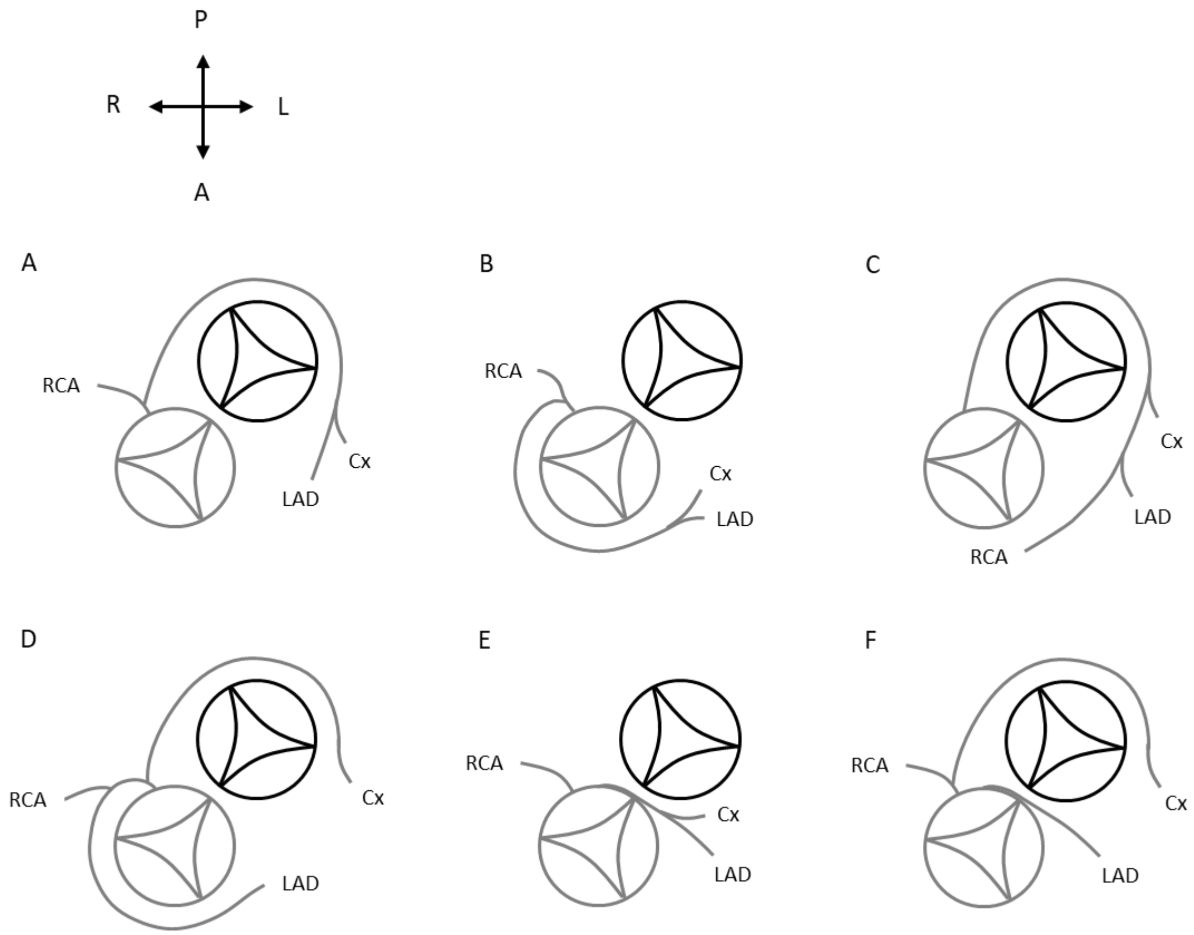

Supplementary Figure 4. Origin, course, and branching pattern of coronary arteries originating from sinus 2 only: A) 2LCxR retropulmonary LCA (n=13); B) 2RLCx anteroaortic LCA (n=2); C) 2RLCx retropulmonary common trunk (n=1); D) 2CxRL retropulmonary Cx, anteroaortic LAD (n=1); E) 2LCx,2R interarterial/intramural LCA (n=19); F) 2L,2CxR interarterial/intramural LAD, retropulmonary Cx (n=5).

Cx, circumflex coronary artery; LAD, left anterior descending coronary artery; LCA, left coronary artery; RCA, right coronary artery.

A

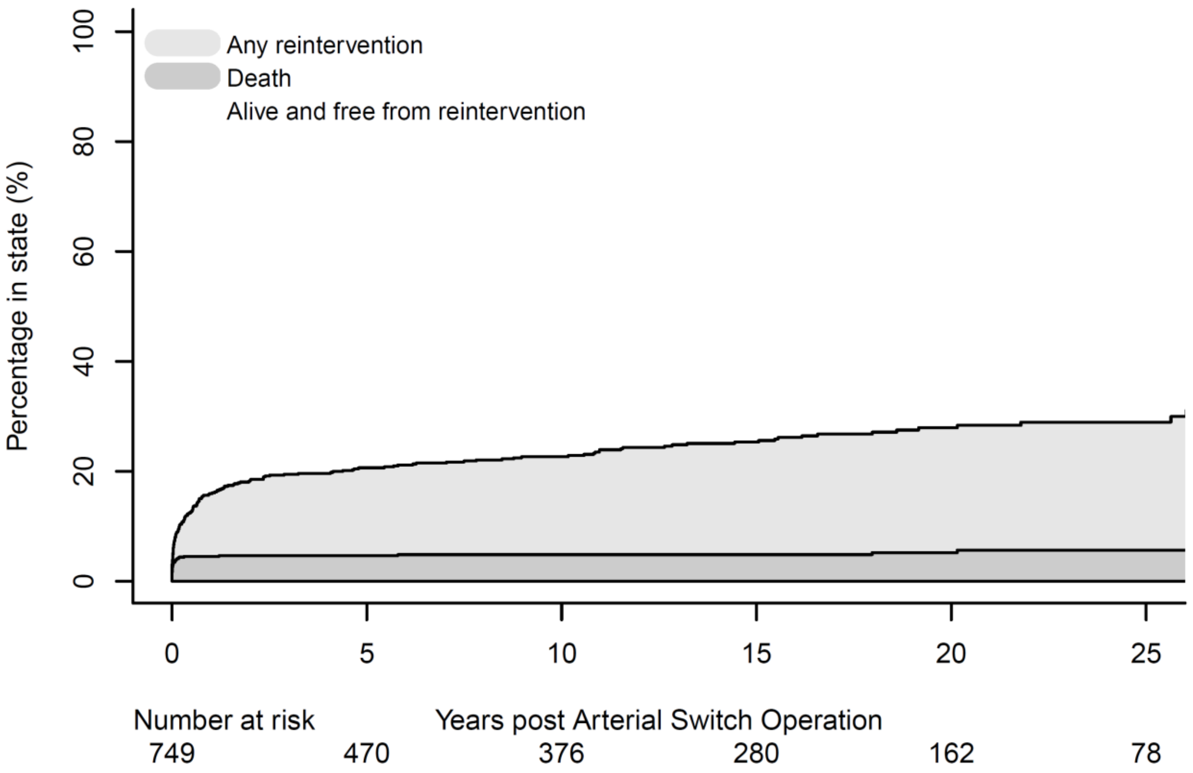
Any reintervention
Death
Alive and free from reintervention
Percentage in state (%)
0
20
40
60
80
100
0
5
10
15
20
25
Number at risk
Years post Arterial Switch Operation
749
470
376
280
162
78

B

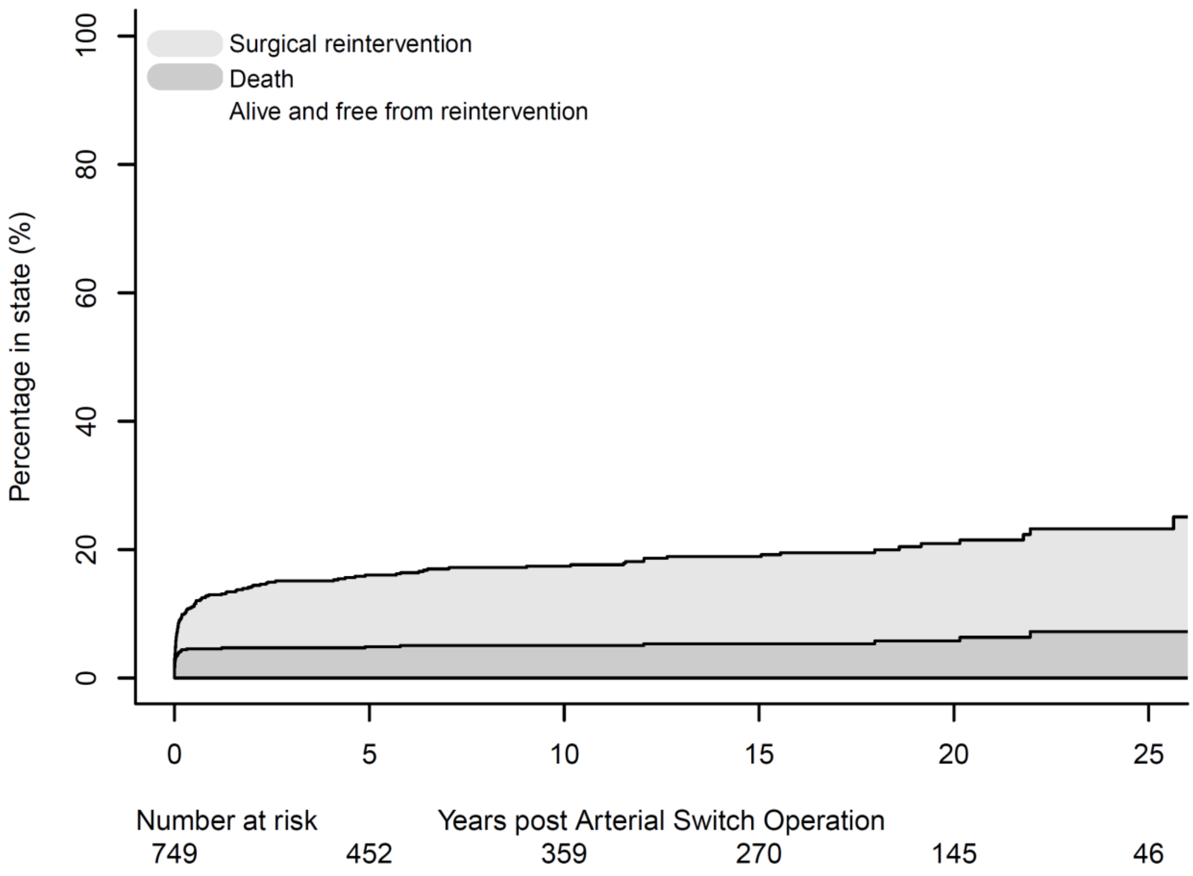
Surgical reintervention
Death
Alive and free from reintervention
Percentage in state (%)
0
20
40
60
80
100
0
5
10
15
20
25
Number at risk
Years post Arterial Switch Operation
749
452
359
270
145
46

C

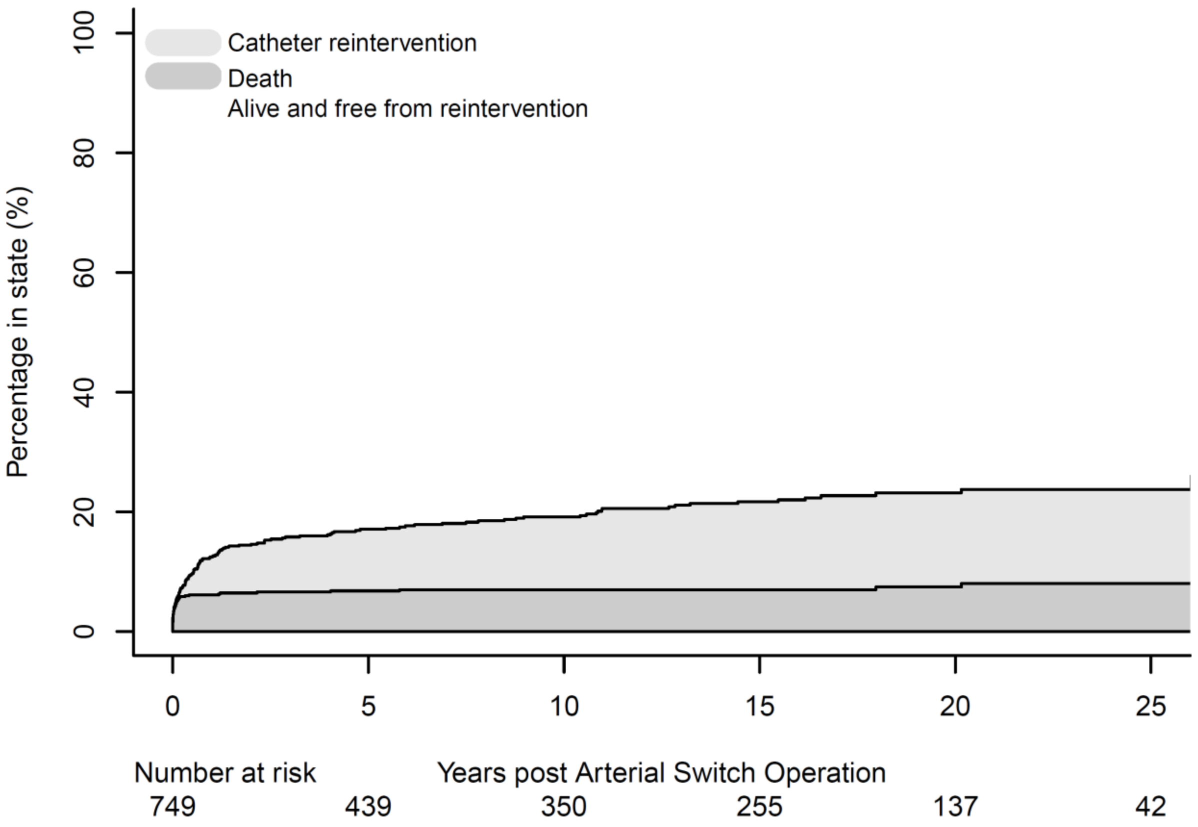

Supplementary Figure 5. Cumulative incidence function plots for A) any reintervention, B) surgical reintervention, and C) catheter reintervention following arterial switch.

A

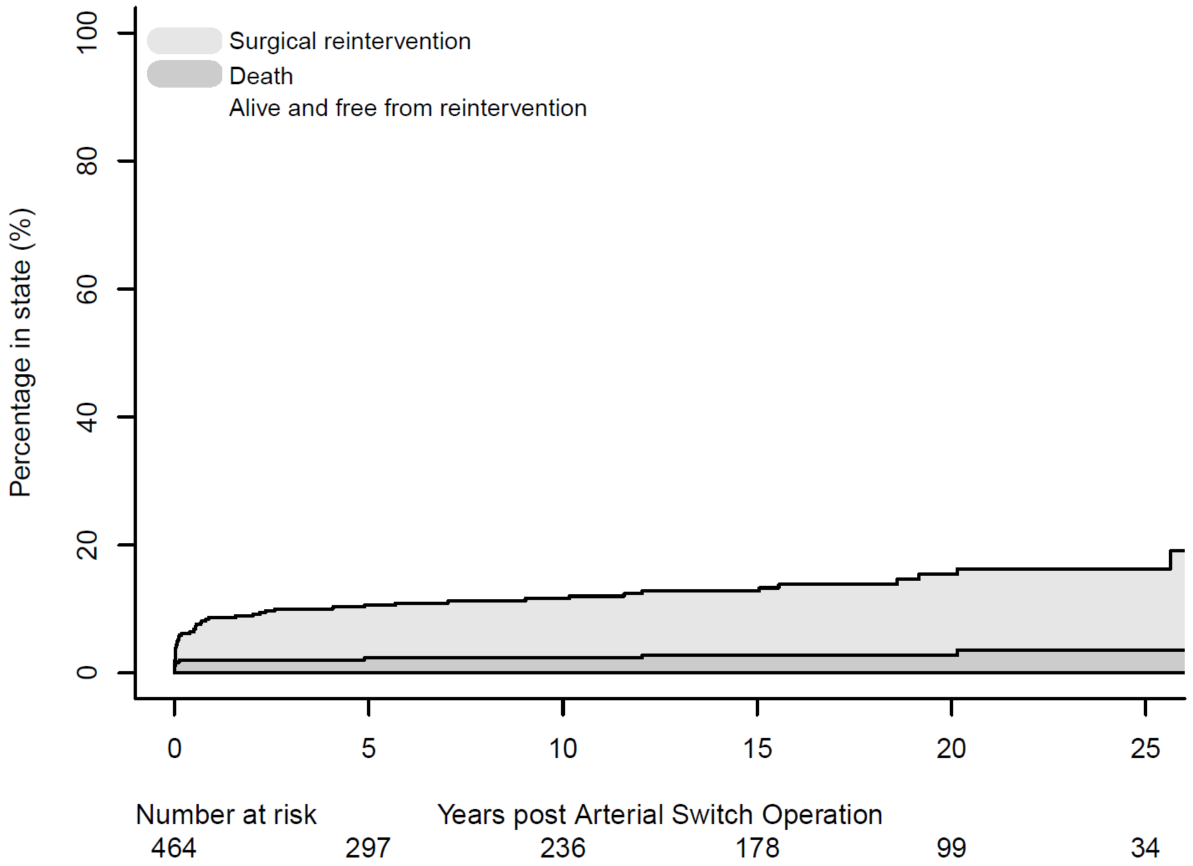
Surgical reintervention
Death
Alive and free from reintervention
Percentage in state (%)
0
20
40
60
80
100
0
5
10
15
20
25
Years post Arterial Switch Operation
Number at risk
464
297
236
178
99
34

B

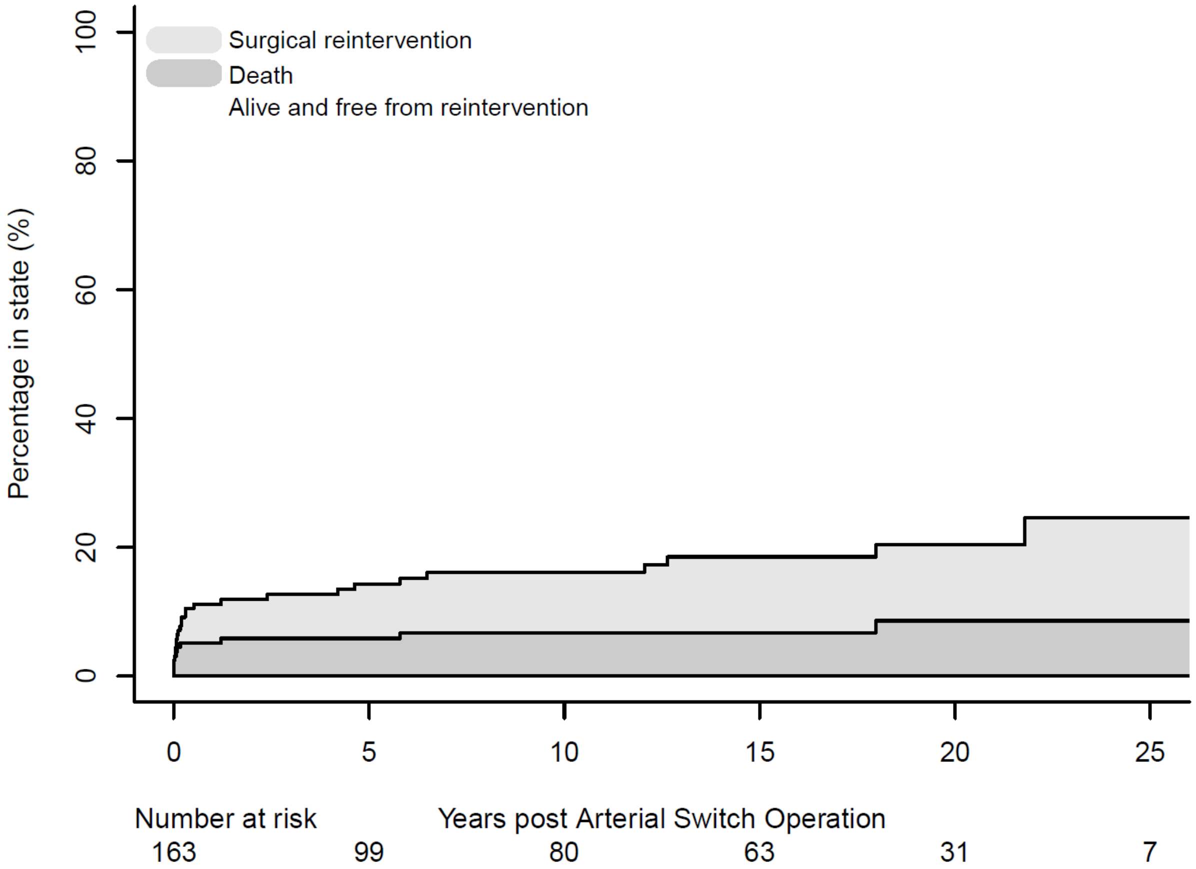
Surgical reintervention
Death
Alive and free from reintervention
Percentage in state (%)
0
20
40
60
80
100
0
5
10
15
20
25
Years post Arterial Switch Operation
Number at risk
163
99
80
63
31
7

C

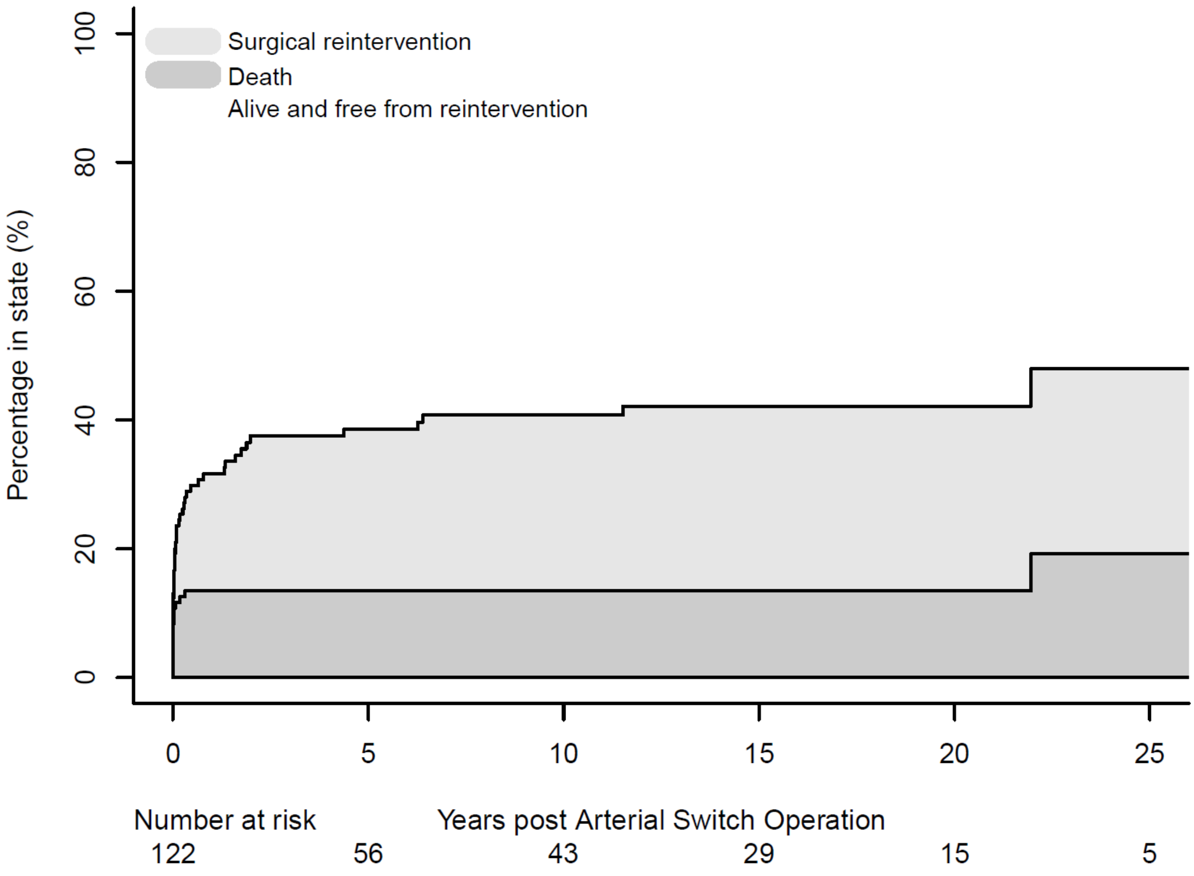

Supplementary Figure 6. Cumulative incidence function plots for surgical reintervention following arterial switch, according to morphological group: A) transposition with intact ventricular septum (TGA-IVS, n=464), B) transposition with a ventricular septal defect (TGA-VSD. N=163), or C) complex transposition with associated anomalies (n=122).

A

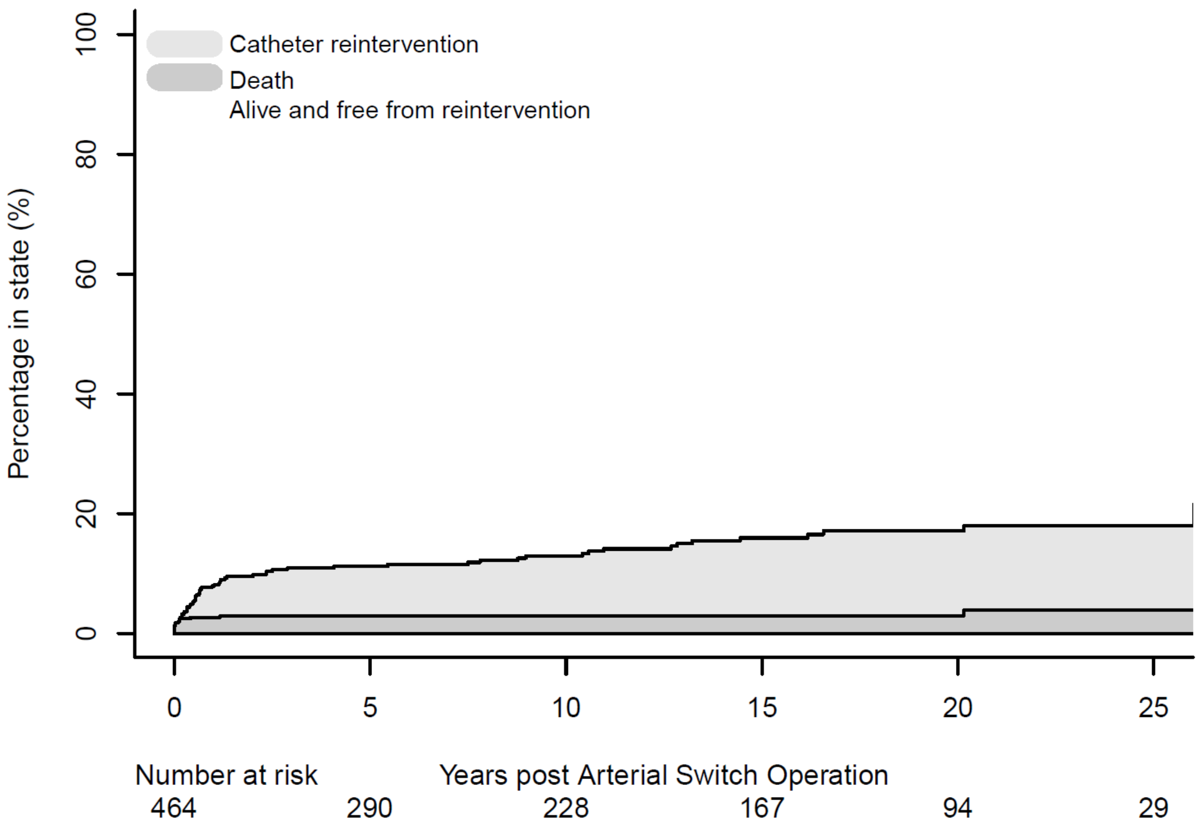
Catheter reintervention
Death
Alive and free from reintervention
Percentage in state (%)
0
20
40
60
80
100
0
5
10
15
20
25
Number at risk
Years post Arterial Switch Operation
464
290
228
167
94
29

B

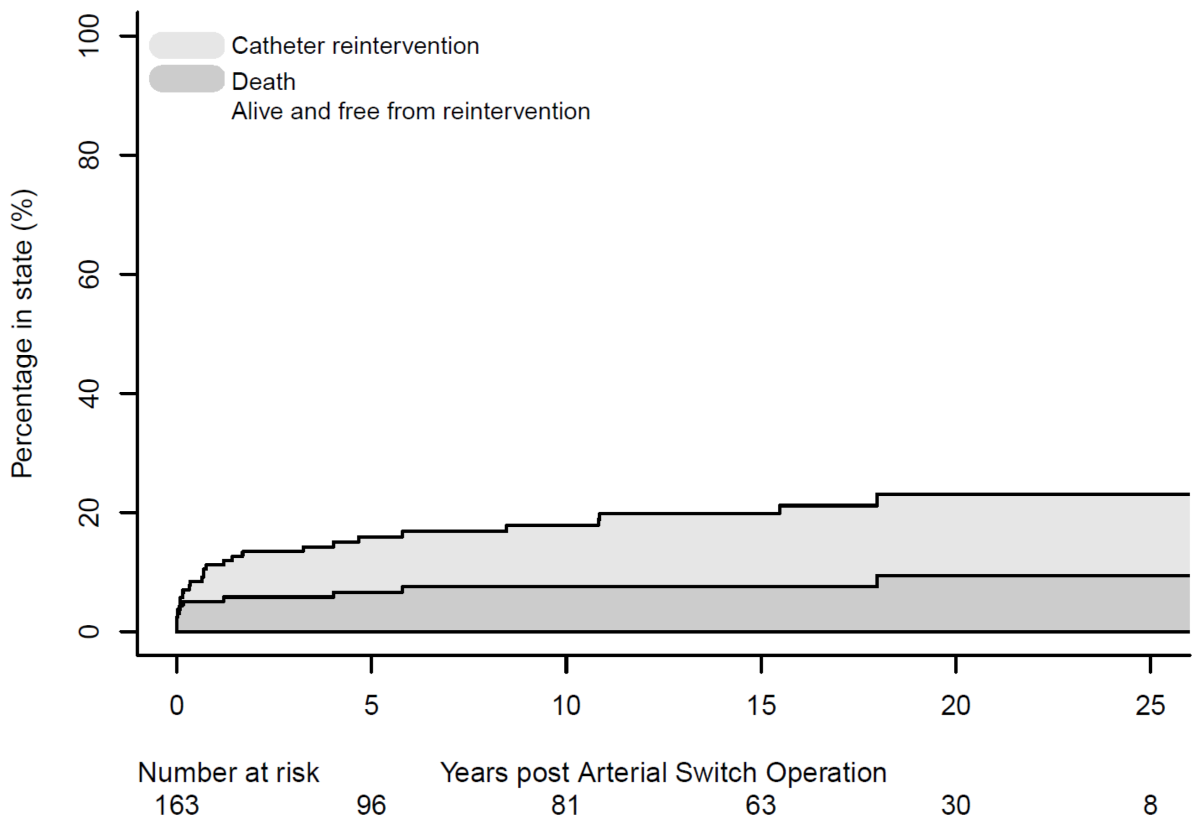
Catheter reintervention
Death
Alive and free from reintervention
Percentage in state (%)
0
20
40
60
80
100
0
5
10
15
20
25
Number at risk
Years post Arterial Switch Operation
163
96
81
63
30
8

C

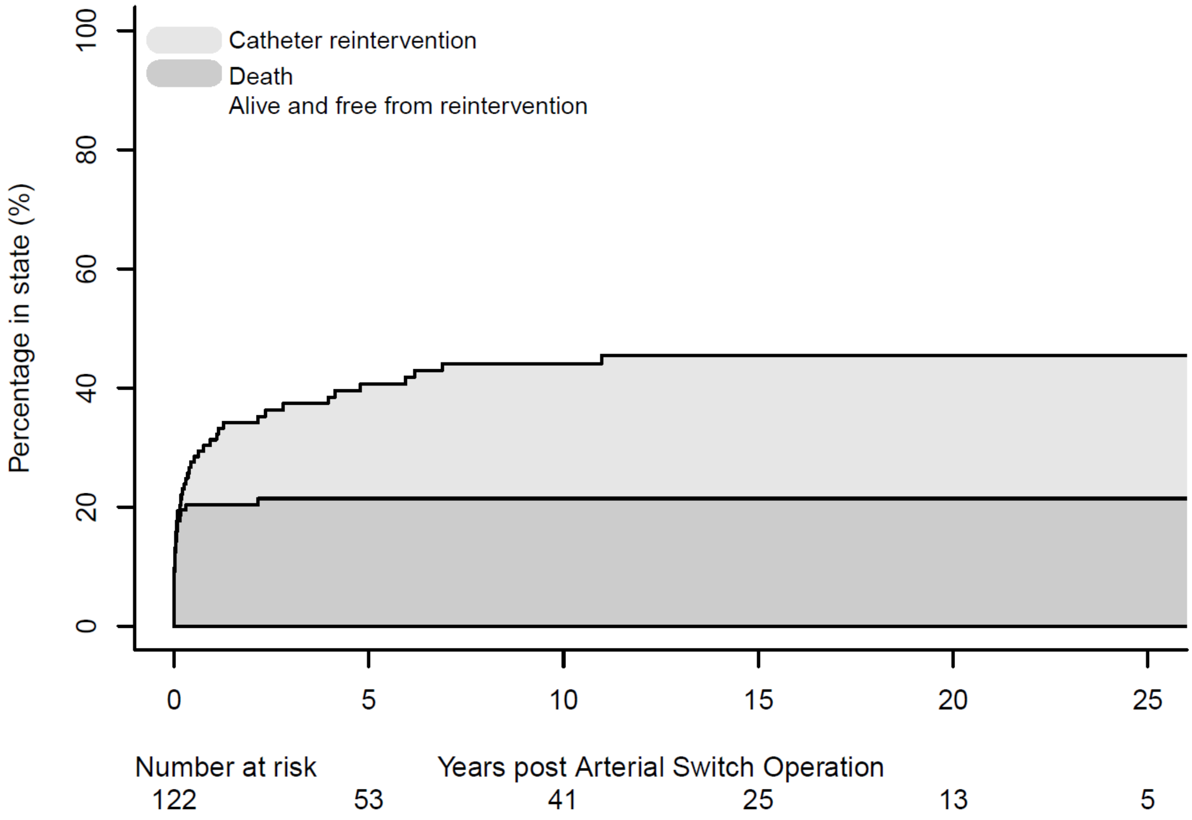

Supplementary Figure 7. Cumulative incidence function plots for catheter reintervention following arterial switch, according to morphological group: A) transposition with intact ventricular septum (TGA-IVS, n=464), B) transposition with a ventricular septal defect (TGA-VSD, n=163), or C) complex transposition with associated anomalies (n=122).
